# Highly efficient MOF-808/polyethersulfone mixed matrix membranes for methyl orange filtration

**DOI:** 10.1039/d6ra07189a

**Published:** 2026-10-19

**Authors:** Xiao-Jing Hu, Qi-Qiang Li, Li-Jie Lin, Xiao-Hui Huang, Shao-Ming Ying

**Affiliations:** a College of New Energy and Materials, Fujian Provincial Key Laboratory of Featured Materials in Biochemical Industry, Ningde Normal University Ningde Fujian 352100 China 11429721@qq.com yingshaoming@126.com

## Abstract

The development of free-standing membranes with high efficiency and robust environmental tolerance remains a key challenge in dye wastewater treatment. Herein, MOF-808-incorporated polyethersulfone (PES) mixed matrix membranes (MMMs) were fabricated for the adsorptive removal of methyl orange (MO). The optimized composite membrane (MOF@PES-2) achieved a maximum MO removal efficiency of 98%. Notably, the membrane demonstrated good salt tolerance, maintaining removal efficiencies of >95% and >89% in MO solutions with NaCl mass concentrations equal to and ten times that of MO, respectively. These results highlight the potential of MOF@PES-2 for efficient dye removal from saline wastewater.

## Abstract 2

The development of free-standing membranes with high efficiency and robust environmental tolerance remains a key challenge in dye wastewater treatment.

## Graphical abstract

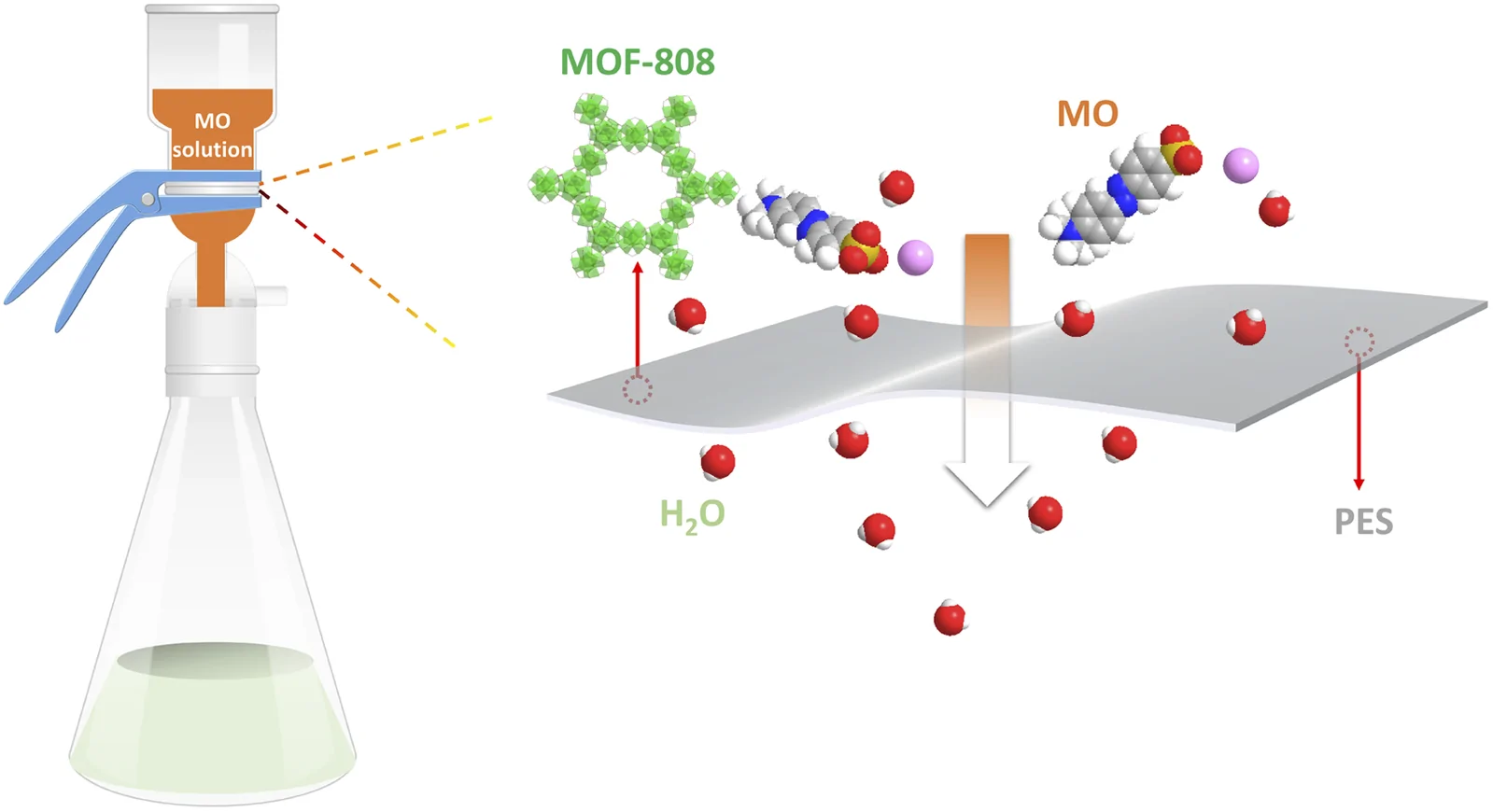

## Introduction

Methyl orange is a common azo dye widely used in industries such as textiles, printing and dyeing, papermaking, and chemicals. However, the substantial discharge of this type of dye during production and use has caused serious pollution to surface water and the ecological environment.^1^ Studies have shown that methyl orange has high chemical stability and is difficult to degrade naturally. Long-term exposure or entry into the human body *via* water may lead to serious health issues such as cell mutation and cancer.^2,3^ Therefore, the efficient removal of methyl orange from aqueous solutions is an important issue concerning both human health and the sustainable development of the ecological environment.

The primary methods currently employed for the removal of methyl orange from water include the following: Photodegradation, which involves the decomposition and decolorization of methyl orange through ultraviolet-visible light irradiation, typically facilitated by photocatalytic materials; Physical methods, which utilize coagulants or flocculants to facilitate the aggregation and sedimentation of methyl orange molecules from aqueous solutions; Biodegradation, which relies on specific microorganisms that secrete enzymes capable of degrading and eliminating dye macromolecules; Advanced oxidation and ozonation, which generate potent oxidizing radicals *via* chemical oxidation processes to decompose methyl orange.^4–6^ And electrochemical approaches, which achieve the decolorization, degradation, and mineralization of organic pollutants and dyes through direct oxidation/reduction of pollutants at electrode reactions.^7^ In comparison, the adsorption method, utilizing porous materials such as metal–organic frameworks (MOFs) and their derivatives, offers a relatively simple operational procedure, requires mild conditions, and is easier to implement, which has garnered significant attention in recent years.^8,9^ However, many existing approaches involve the direct dispersion of MOF materials in water for the purposes of adsorption and separation. This practice poses risks of secondary pollution and complicates material recycling.^10,11^

Recently, mixed matrix membranes (MMMs) incorporating MOFs and polymers have emerged as a prominent area of research, capitalizing on the porous characteristics of MOFs alongside the flexibility of polymers.^12,13^ These materials find extensive applications in diverse fields, including gas separation, catalysis, sensing, and permeation.^14,15^ Furthermore, due to their remarkable adsorption and pollutant removal capabilities, coupled with straightforward preparation and operational processes, MOF-based MMMs have been widely employed for the adsorption and removal of contaminants from water. For example, a MMM consisting of Bio-MOF-2Me and polyvinylidene fluoride could still remove 72% of cationic methyl violet even after five cycles.^16^ Similarly, a MMM fabricated from ZIF-67 and polyvinylidene fluoride achieves a removal efficiency of up to 99.5% for Congo red in aqueous solutions.^17^ Collectively, these findings underscore the significant research value of MOF-polymer MMMs in the context of water pollution remediation.

In this work, MOF-808, a classic zirconium-based metal–organic framework (MOF) featuring outstanding hydrolytic stability and hierarchical porosity,^18,19^ was systematically evaluated for the adsorptive removal of methyl orange (MO) from aqueous solutions. Compared with other Zr-MOFs such as UiO-66 and MOF-801, MOF-808 possesses a more open framework with larger pore apertures and a higher specific surface area,^20,21^ which are particularly beneficial for the adsorption of bulky dye molecules and the fabrication of mixed matrix membranes. To facilitate practical environmental applications, a free-standing polyethersulfone (PES) mixed matrix membrane was successfully fabricated. PES was chosen as the host matrix owing to its excellent film-forming properties and chemical robustness,^22,23^ while the MOF-808/PES combination offers structural synergy—PES provides mechanical support and the MOF contributes high porosity and hydrophilicity. The chemical compatibility arising from potential hydrogen bonds and π–π stacking further ensures uniform filler dispersion within the membrane.^24^ Experimental results demonstrate that, when employed as a continuous filtration medium, this membrane achieves a maximum MO removal efficiency of over 98%. Furthermore, even in a binary solution containing sodium chloride (NaCl) at an equivalentmass concentration to MO, the membrane sustained an outstanding removal efficiency exceeding 95%, exhibiting excellent resistance to salt interference (Scheme 1).

**Scheme 1:**
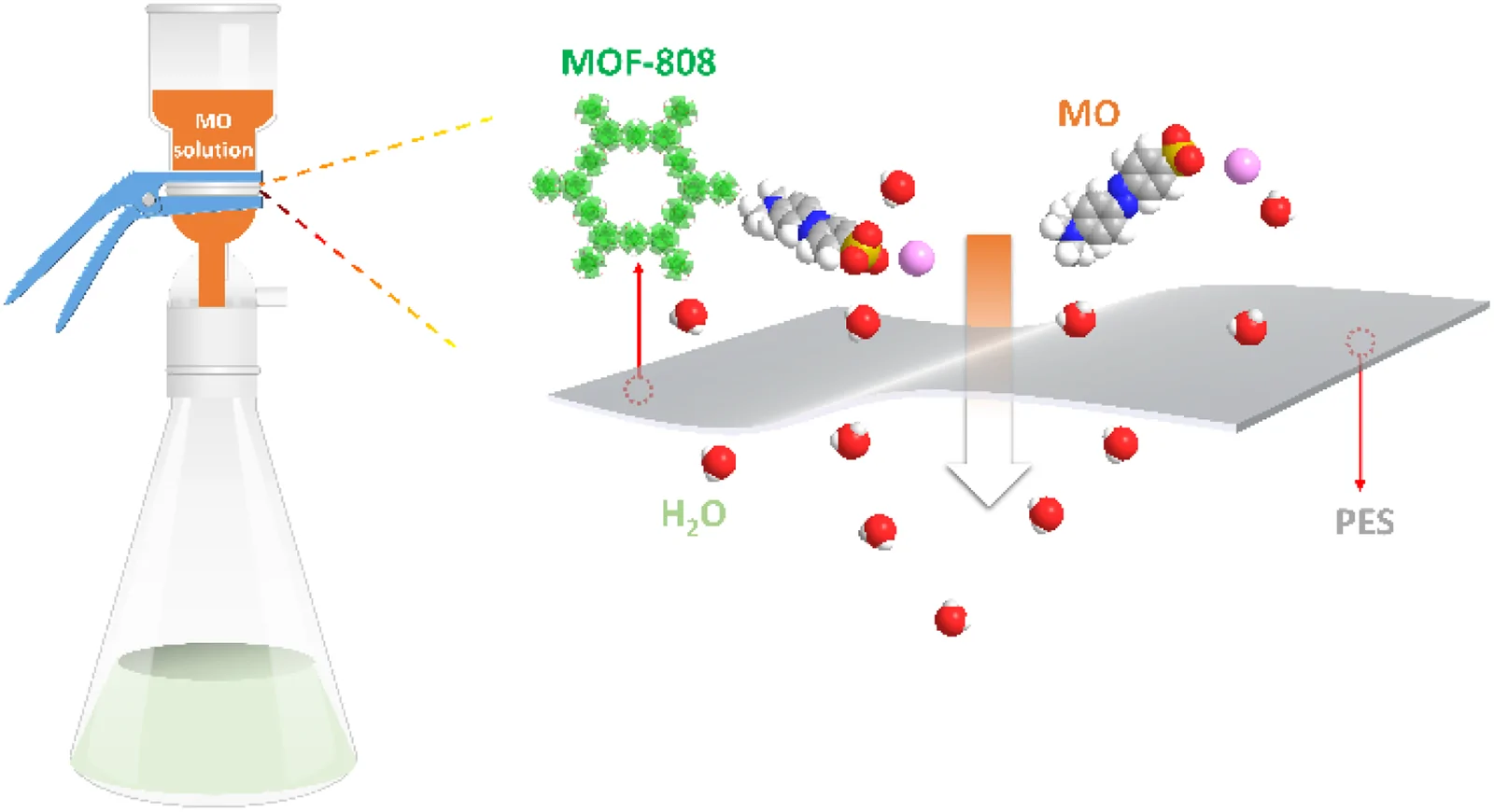
Schematic diagram illustrating the MO removal process by the MOF-808-loaded polyethersulfone membrane *via* facile filtration.

## Experimental section

### Synthesis and characterization of MOF-808

Details of the preparation and characterization of the MOF-808 are described in the SI.

### MO adsorption experiments of MOF-808

Details of the adsorption kinetics experiment and adsorption isotherm experiment are provided in the SI.

### Synthesis of membranes

MOF-based MMMs, denoted as MOF@PES-1 and MOF@PES-2, were fabricated by blending MOF-808 with polyethersulfone *via* a simple solution-casting method. Typically, MOF-808 nanoparticles (0.45 g for MOF@PES-1 and 0.75 g for MOF@PES-2) and PES (1.5 g) were dispersed in 9 mL of DEF under stirring for 12 h to form a uniform casting dope. The mixture was then poured onto a flat glass plate and cast using a doctor blade. After air-drying at room temperature for a few minutes, the membrane was sprayed with industrial-grade ethanol and detached from the glass substrate, yielding a free-standing film. To systematically evaluate the MO removal performance of these MMMs, a pristine PES membrane was also fabricated under identical experimental conditions to serve as a control.

### MO removal experiments of membranes

A vacuum filtration apparatus was employed to evaluate the MO removal performance of the membranes. For each test, the membrane was sandwiched between two pieces of filter paper, clamped in the filtration cell, and pre-flushed thrice with ultrapure water before being used for MO filtration.

The MO removal efficiency (%), defined as the change in MO concentration before and after a single filtration run, was employed to evaluate the performance of the fabricated membranes, and was calculated using the following equation:1
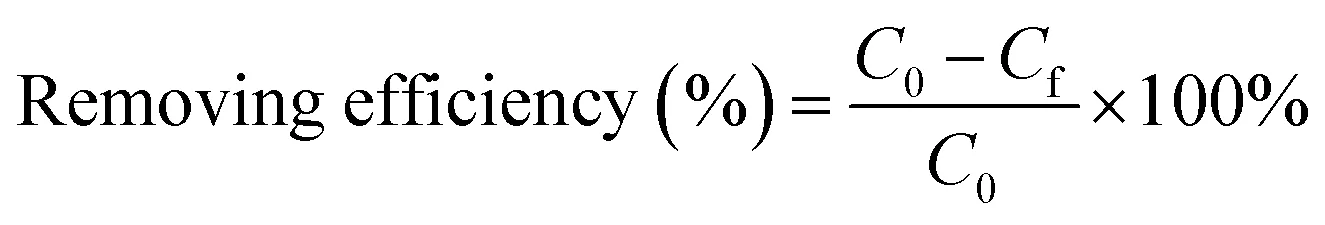
where *C*_0_ (mg L^−1^) is the initial concentration, *C*_f_ (mg L^−1^) is the concentration after once filtration.

## Results and discussion

### Characterization of MOF-808 samples

Initially, pristine MOF-808 powder was synthesized, and its crystalline structure was verified *via* powder X-ray diffraction (PXRD, Fig. S1). The diffraction peaks of the as-synthesized sample match perfectly with the theoretically simulated pattern,^25^ confirming the successful formation of highly pure MOF-808. Furthermore, the nitrogen adsorption isotherms at 77 K (Fig. S2) aligns seamlessly with previously reported literature,^25^ with a BET surface area of 2064 m^2^ g^−1^, verifying its well-developed porous structure. And The thermal behaviour of the obtained MOF-808 sample is in good agreement with that reported for pristine MOF-808,^26^ confirming the good thermal stability of the synthesized MOF-808 up to approximately 200 °C (Fig. S3). Morphologically, scanning electron microscopy (Fig. S4) reveals that the MOF-808 particles exhibit a well-defined regular octahedral shape, which is highly consistent with established data.^27,28^ Additionally, dynamic light scattering analysis (Fig. S5) demonstrates a narrow size distribution with an average particle diameter of approximately 200 nm. This uniform nanoscale dimension is highly advantageous for the homogeneous dispersion of MOF particles during the subsequent fabrication of mixed matrix membranes.

### MO adsorption studies of MOF-808

To evaluate the intrinsic adsorption performance of pristine MOF-808 toward MO, batch adsorption experiments were performed using MOF-808 powder (*C*_0_ = 30 mg L^−1^, *V* = 100 mL, *m*_MOF-808_ = 20 mg). As illustrated in Fig. 1a, the adsorption of MO proceeded rapidly during the initial ten minutes, achieving an uptake capacity of 64.99 mg g^−1^. Over the subsequent 10 to 40 minutes, the capacity steadily increased to 107.23 mg g^−1^. Thereafter, the adsorption rate decelerated as the system gradually approached equilibrium, ultimately reaching an equilibrium capacity of 132.19 mg g^−1^ within 240 minutes. These results demonstrate the favourable kinetics and a comparable MO capture capability of the MOF-808 powder.^29–31^ To further elucidate the adsorption behaviour, the adsorption kinetics of MOF-808 toward MO were investigated by fitting the adsorption data with pseudo-first-order and pseudo-second-order kinetic models (eqn. S2 and S3). The corresponding fitting curves and parameters are summarized in Fig. 1a and Table S1. Obviously, the adsorption process is significantly better described by the pseudo-second-order model (*R*_2_ = 0.9997, compared to 0.982 for the pseudo-first-order model), suggesting that the adsorption rate is governed by the accessible active sites on the adsorbent surface, and the process is likely dominated by chemisorption or strong electrostatic interactions. Based on this model, the calculated equilibrium adsorption capacity (*Q*_e_) was 138.96 mg g^−1^, which aligns closely with the experimental capacity achieved within 240 minutes (*Q*_240min_, 132.19 mg g^−1^).

**Fig. 1:**
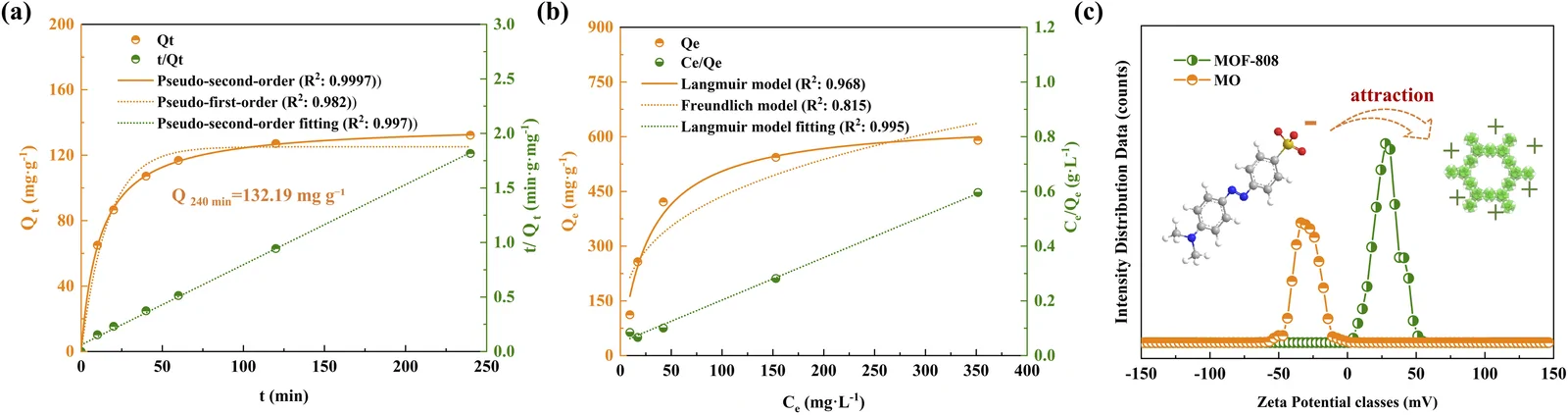
(a) Adsorption kinetics of MO on MOF-808; (b) adsorption isotherms of MO on MOF-808; (c) zeta potentials of MO and MOF-808.

In addition, the adsorption isotherms, conducted with 10 mg adsorbent and initial MO concentrations ranging from 0 to 400 mg L^−1^, were analyzed by employing the Langmuir and Freundlich models (eqn. S4 and S5; Fig. 1b and Table S2). Obviously, the experimental data exhibit a better fit to the Langmuir model (*R*_2_ = 0.968) than to the Freundlich model (*R*_2_ = 0.815), suggesting monolayer adsorption on a relatively homogeneous surface. Derived from the Langmuir equation, the theoretical maximum adsorption capacity (*Q*_max_) of MOF-808 for MO reaches an impressive 646.98 mg g^−1^, which not only substantially outperforms conventional commercial activated carbon (129.3 mg g^−1^) but also exceeds most Zr-MOFs listed in Table S3.^32^ These results suggest that MOF-808 is a promising adsorbent for MO removal. Considering that MO contains a sulfonate group that is negatively charged in aqueous solution, and that the surface of MOF-808 is likely positively charged due to coordinatively unsaturated zirconium sites, strong electrostatic attraction may exist between MO and the MOF-808 surface, thereby promoting the efficient adsorption and removal of MO. To verify this hypothesis, zeta potential measurements were conducted on both MOF-808 and the aqueous MO solution (Fig. 1c). The results clearly indicate that the MO solution carries a distinct negative charge, whereas MOF-808 exhibits a strongly positive surface charge. This profound electrostatic affinity is likely the primary driving force behind the outstanding MO removal performance of the MOF.

To confirm that adsorption is primarily driven by electrostatic attraction, pH-dependent adsorption experiments were performed (*C*_0_ = 30 mg L^−1^, *V* = 100 mL, *m*_MOF-808_ = 20 mg). As shown in Fig. 2a, the adsorption of methyl orange (MO) on MOF-808 exhibited a pronounced pH dependence. The adsorption capacity reached a maximum of approximately 140 mg g^−1^ at pH 4 and decreased sharply under strongly acidic (pH 2) and strongly alkaline (pH 12) conditions. Notably, in our previous report, MOF-808 was shown to exhibit excellent water stability over a wide pH range in aqueous solutions, ruling out the possibility that the sharp decrease in adsorption capacity at extreme pH values arises from structural degradation of the framework.^33^ The correlation between the zeta potential of MOF-808 and the p*K*_a_ of MO (∼3.37)^34^ indicates that electrostatic interactions are the dominant driving force. At pH 4, the positively charged MOF-808 surface and the predominantly deprotonated anionic MO species (MO^−^) generate the strongest electrostatic attraction, resulting in maximum adsorption. At pH 2, protonation of MO to its acidic form (HMO) and charge screening by excess H^+^ suppress MO uptake. At pH 12, the negatively charged MOF-808 surface electrostatically repels MO^−^, leading to a nearly complete loss of adsorption capacity. These results demonstrate that the pH-dependent adsorption behaviour of MOF-808 toward MO is primarily governed by electrostatic interactions.

**Fig. 2:**
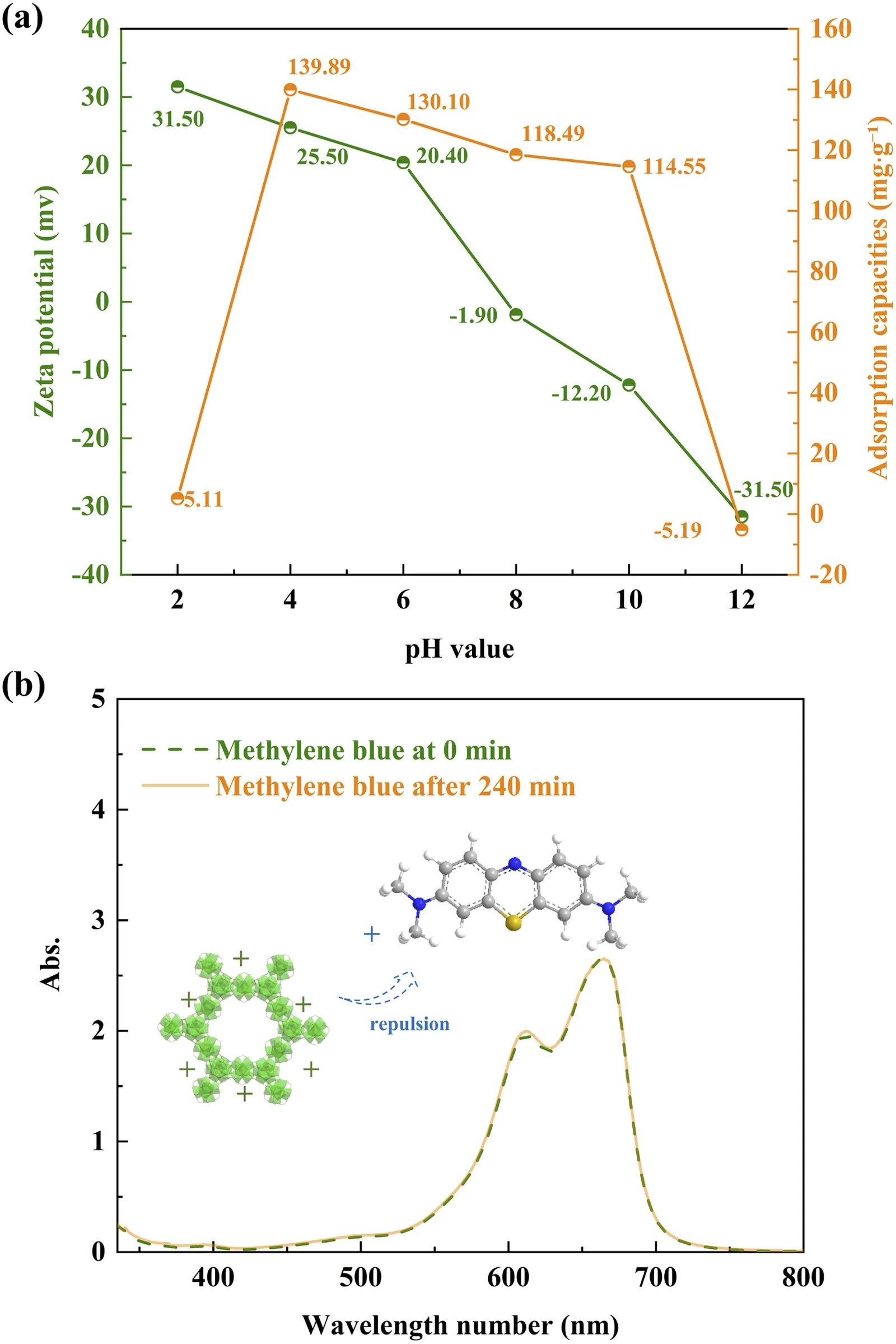
(a) Zeta potentials and MO adsorption capacities of MOF-808 at different pH values (*C*_0_ = 30 mg L^−1^, *V* = 100 mL, *m*_MOF-808_ = 20 mg). (b) UV-vis absorption spectra of the MB solution before (0 min) and after (240 min) contact with MOF-808. Inset: schematic illustration of the electrostatic repulsion between the positively charged MOF-808 surface and the cationic MB molecules.

Furthermore, XPS analysis of MOF-808 before and after MO adsorption was performed. As shown in Fig. S7 and S8, no significant shifts were observed in the Zr 3d and O 1s spectra, indicating that the interaction between the sulfonate groups of MO and the Zr sites is predominantly electrostatic attraction rather than strong chemical coordination. This is consistent with the zeta potential results. Besides, control experiments were carried out under the same conditions using methylene blue (MB), a typical cationic dye. As depicted in Fig. 2b, MOF-808 exhibited virtually no adsorption capacity toward the cationic MB molecules, consistent with the electrostatic repulsion between the positively charged MOF-808 surface and the cationic dye. The combined results strongly support that the adsorption is predominantly driven by electrostatic attraction.

### Characterization of membranes

First, the presence of MOF-808 within the fabricated membranes were confirmed *via* powder X-ray diffraction (Fig. 3a). Notably, the XRD patterns of both mixed matrix membranes, MOF@PES-1 and MOF@PES-2, exhibit the characteristic diffraction peaks of MOF-808. This indicates that MOF-808 successfully retained its intrinsic crystalline framework within the polymer matrix without significant structural alteration. In contrast, the pristine PES membrane exhibited a typical amorphous pattern with no apparent crystallinity. In addition, ATR-FTIR spectra were recorded for pristine MOF-808, the pristine PES membrane, and MMMs with different MOF-808 loadings, as shown in Fig. 3b. All MMMs displayed FTIR patterns highly consistent with those of the pristine PES membrane, indicating that PES is the dominant component of the membrane matrix. Nonetheless, the characteristic peaks of MOF-808 remained clearly discernible in the MMMs. In pristine MOF-808, the band at approximately 1618 cm^−1^ arises from the strong C=C stretching of aromatic rings, while the bands at 1443 and 1379 cm^−1^ are attributed to the symmetric COO stretching modes in combination with other ring deformations. In addition, the peak at approximately 655 cm^−1^ corresponds to the Zr–O vibration of the Zr_6_O_8_ nodes.^35^ Notably, compared with pristine MOF-808, these characteristic peaks in the MMMs exhibit a noticeable blue shift to higher wavenumbers, indicating that the chemical environment of the Zr–O bonds and carboxylate linkers in MOF-808 is altered after being embedded in the PES matrix; we attribute this to the spatial confinement effect of the PES chains and possible weak interfacial interactions, such as hydrogen bonding or coordination, between the Zr–OH sites of MOF-808 and the polar groups (C–O–C or O=S=O) of PES. It is worth noting that the characteristic peaks of the PES backbone do not show significant shifts, suggesting that the interfacial interactions are primarily localized at the MOF surfaces without substantially altering the bulk chemical structure of PES, which is beneficial for maintaining the intrinsic membrane flexibility, improving the compatibility between MOF-808 and PES, and restricting polymer chain mobility.

**Fig. 3:**
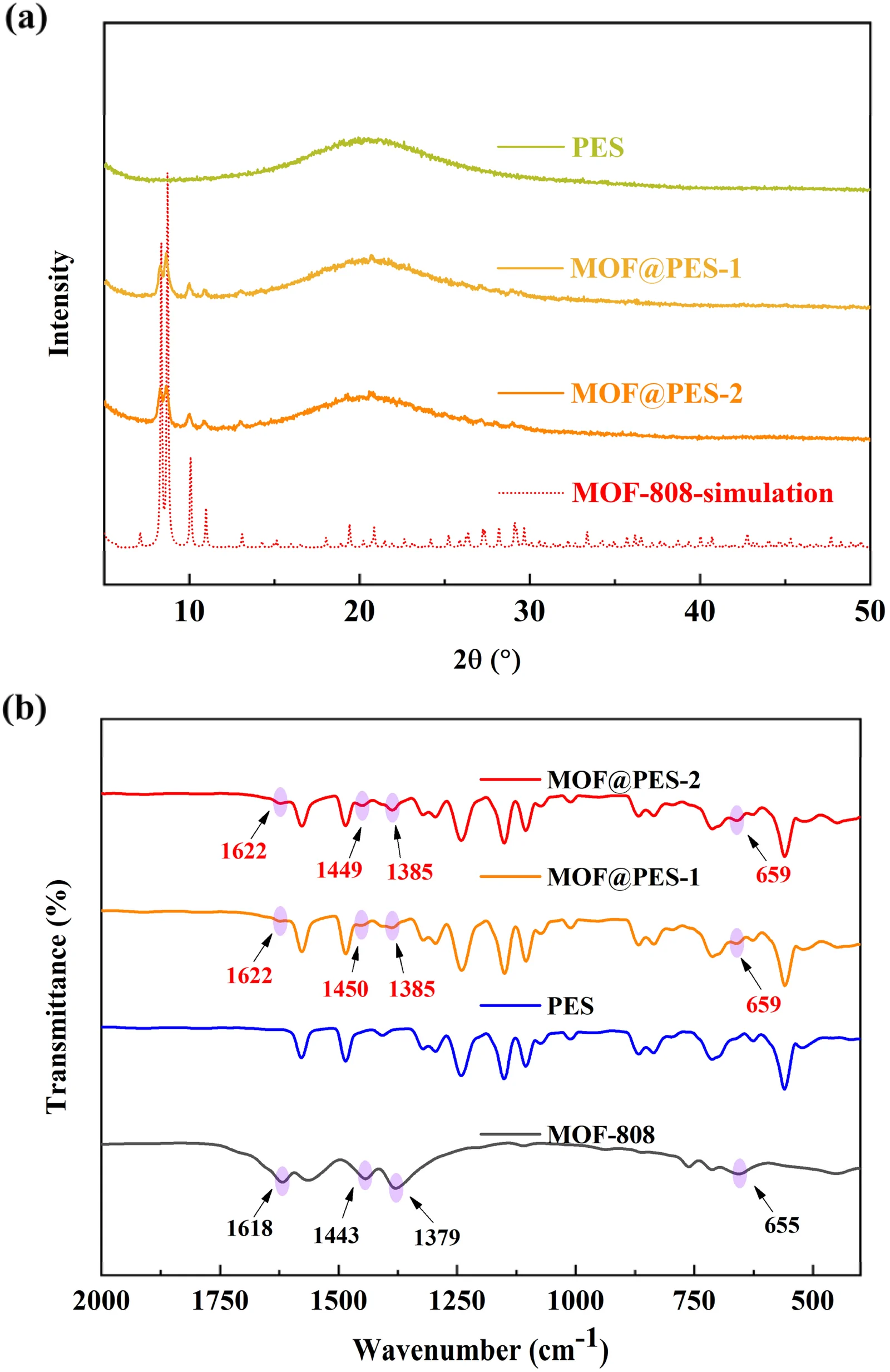
(a) PXRD patterns of the obtained membranes. (b) ATR-FTIR spectra of pristine MOF-808, the pristine PES membrane, and MMMs.

The surface and cross-sectional morphologies of the fabricated membranes were also investigated using scanning electron microscopy (SEM). As illustrated in Fig. 4a and S10, the pristine PES membrane exhibits a relatively smooth surface, whereas the surfaces of MOF@PES-1 and MOF@PES-2 display noticeable roughness and are densely populated with MOF-808 microparticles. All membranes possess abundant surface pores, which minimize hydraulic resistance and facilitate permeation of aqueous solutions. The cross-sectional SEM images (Fig. 4b and S11) also reveal a loose and highly porous internal architecture. Furthermore, the membrane thickness was determined from the cross-sectional SEM images, and the data are presented as the mean ± standard deviation of three measurement points on a single membrane for each material. The mean thicknesses were 23.72 ± 0.98, 30.28 ± 0.63, and 33.79 ± 0.70 µm for pristine PES, MOF@PES-1, and MOF@PES-2, respectively. These results suggest that MOF incorporation increases the membrane thickness under the tested conditions. EDS mapping (Fig. S12 and S13) further confirms the existence of Zr signals from MOF-808 across both surfaces and cross-sections. Furthermore, to quantify the actual MOF-808 loading in the MMMs, inductively coupled plasma optical emission spectrometry (ICP-OES) was employed to determine the zirconium content. As shown in Table S4, the pristine MOF-808 powder contained 38.32 wt% Zr. Based on this value, the actual MOF-808 loadings were calculated to be 15.37 wt% for MOF@PES-1 (Zr: 5.89 wt%) and 28.29 wt% for MOF@PES-2 (Zr: 10.84 wt%), which are slightly lower than the theoretical values of 23.08 wt% and 33.33 wt%, respectively. This deviation is commonly observed in MMM fabrication and attributed to the partial loss of MOF-808 particles during the non-solvent induced phase inversion process, such as leaching into the coagulation bath or incomplete dispersion in the casting solution. Nevertheless, the actual MOF loading increases proportionally with the feed ratio, confirming the successful and controllable incorporation of MOF-808 into the PES matrix.

**Fig. 4:**
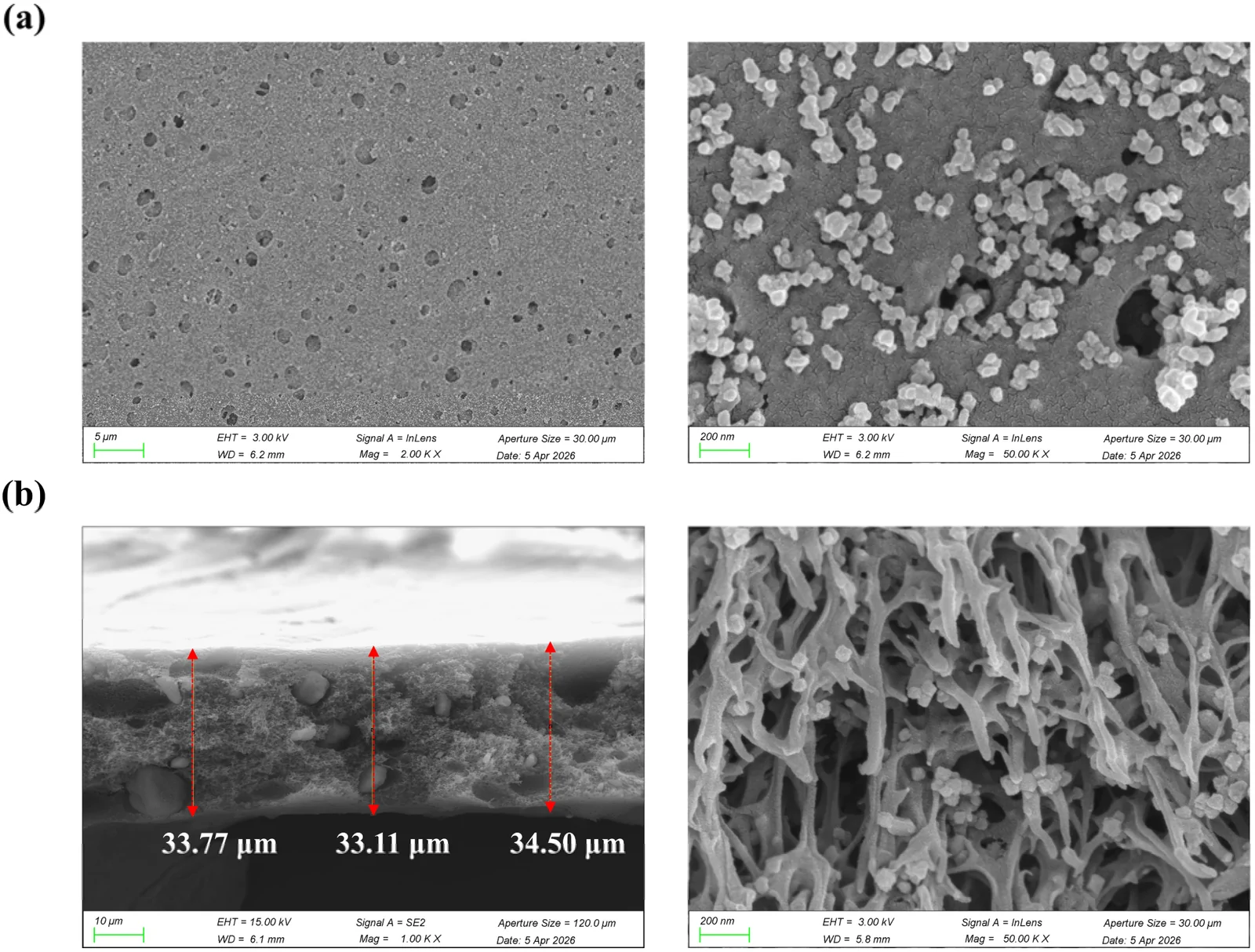
(a) Surface and (b) cross-sectional SEM images of MOF@PES-2.

Meanwhile, mercury intrusion porosimetry (MIP) was employed to quantify the porous architecture of the membranes. As shown in Fig. 5a, the pristine PES membrane exhibited the highest porosity of 75.17%. Upon MOF incorporation, the porosity decreased to 69.59% for MOF@PES-1, likely due to partial pore blockage by MOF particles. At a higher loading (MOF@PES-2), the porosity recovered to 73.79%, which is attributed to the formation of interparticle voids among aggregated MOF nanoparticles. The pore size distributions in Fig. 5b show that PES and MOF@PES-1 exhibit similar profiles, with pore sizes mainly in the 20∼2000 nm range and a main peak centred at approximately 300 nm. In contrast, the pore size distribution of MOF@PES-2 is mainly in the range of 100–2000 nm, with the predominant peak shifted to approximately 700 nm, indicating that a higher MOF loading enlarges the pore size. These quantitative results are supporting the highly porous architecture and successful incorporation of MOF-808.

**Fig. 5:**
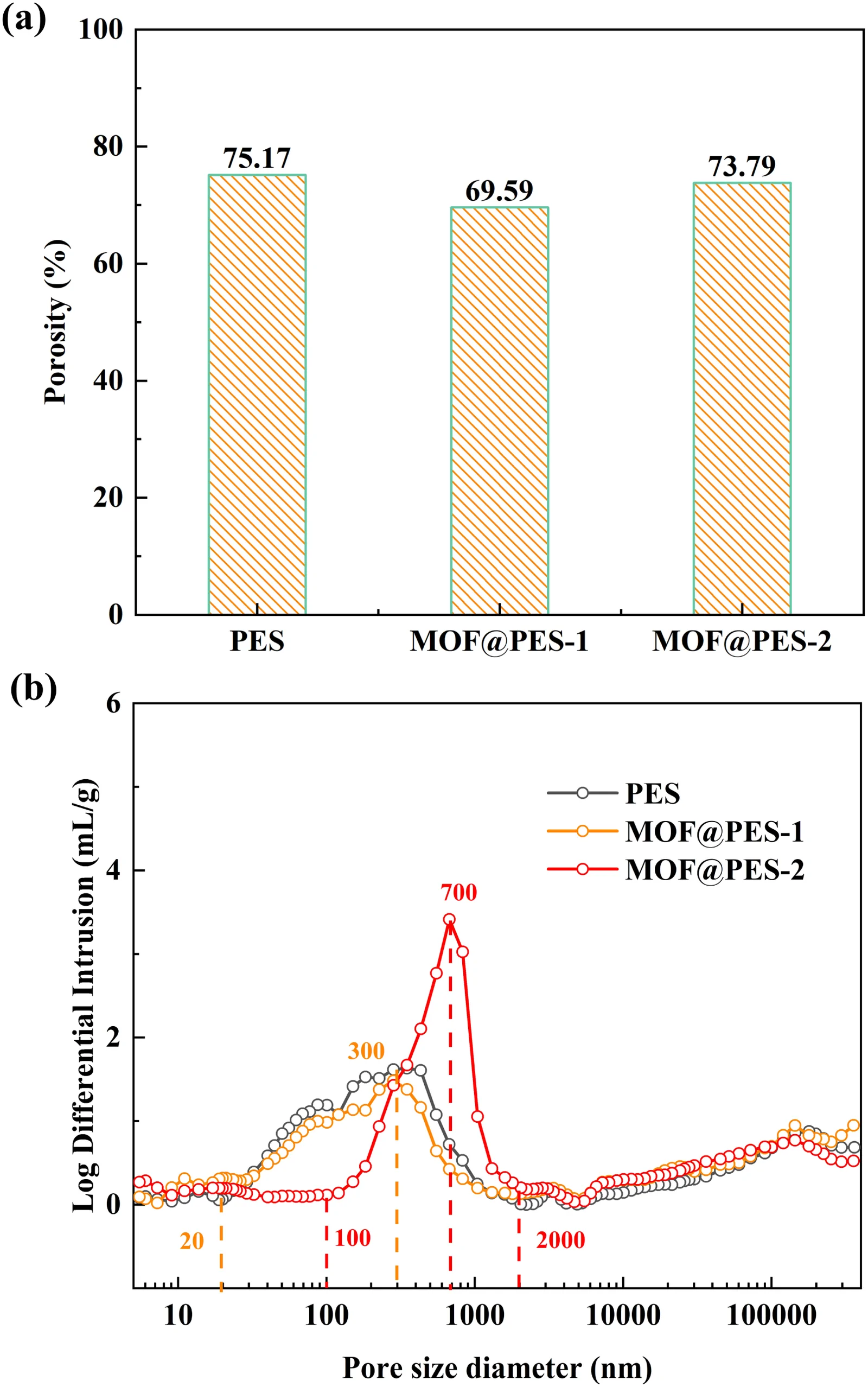
(a) Pore size distribution and (b) porosity of the obtained membranes, as determined by MIP.

Subsequently, the mechanical properties of the membranes were evaluated using an electronic universal testing machine. For each membrane sample, three parallel tests were performed. The average values of the measured parameters are presented in Fig. 6, and the detailed experimental data are provided in Tables S5–S7. First, the tensile strength increased slightly from the pristine PES membrane to MOF@PES-1, with values of 14.86 ± 1.22 and 16.09 ± 2.44 MPa, respectively. However, it decreased sharply to 2.83 ± 0.08 MPa for MOF@PES-2. This indicates that a low MOF loading can slightly enhance the tensile strength, but an excessive MOF loading severely weakens the mechanical strength, likely due to MOF aggregation and the formation of structural defects within the membrane. For elongation at break, the pristine PES membrane had the highest value (12.53 ± 6.40%), while MOF@PES-1 decreased notably to 6.34 ± 1.17%. MOF@PES-2 showed a value of 10.65 ± 1.99%, which was still lower than that of the pristine PES membrane but higher than that of MOF@PES-1. This suggests that the incorporation of MOF generally reduces the flexibility of the membrane, and although the elongation at break increases again at a higher MOF loading, it remains lower than that of the pristine PES membrane, possibly related to increased structural heterogeneity within the membrane. Regarding the elastic modulus, MOF@PES-1 showed a slight decrease (366.91 ± 86.37 MPa) compared with the pristine PES membrane (390.12 ± 57.82 MPa), whereas MOF@PES-2 exhibited a substantial increase to 527.84 ± 182.63 MPa. Notably, the standard deviation of the elastic modulus was relatively large for all membranes and increased progressively with MOF loading (57.82, 86.37, and 182.63 MPa for pristine PES, MOF@PES-1, and MOF@PES-2, respectively), indicating that sample-to-sample heterogeneity became more pronounced as the MOF content increased. Despite the large standard deviation in the elastic modulus of MOF@PES-2, likely due to the intrinsic heterogeneity of the membrane, the overall increasing trend in elastic modulus suggests that the introduction of MOF-808 increases membrane stiffness, which is highly beneficial for mitigating pressure-induced pore compaction during practical water treatment operations. However, the progressively increasing standard deviation of the elastic modulus also suggests that a higher MOF content may aggravate membrane heterogeneity. Therefore, further increasing the MOF/PES feed ratio is not advisable, as it may exacerbate the heterogeneity of the resulting membranes.

**Fig. 6:**
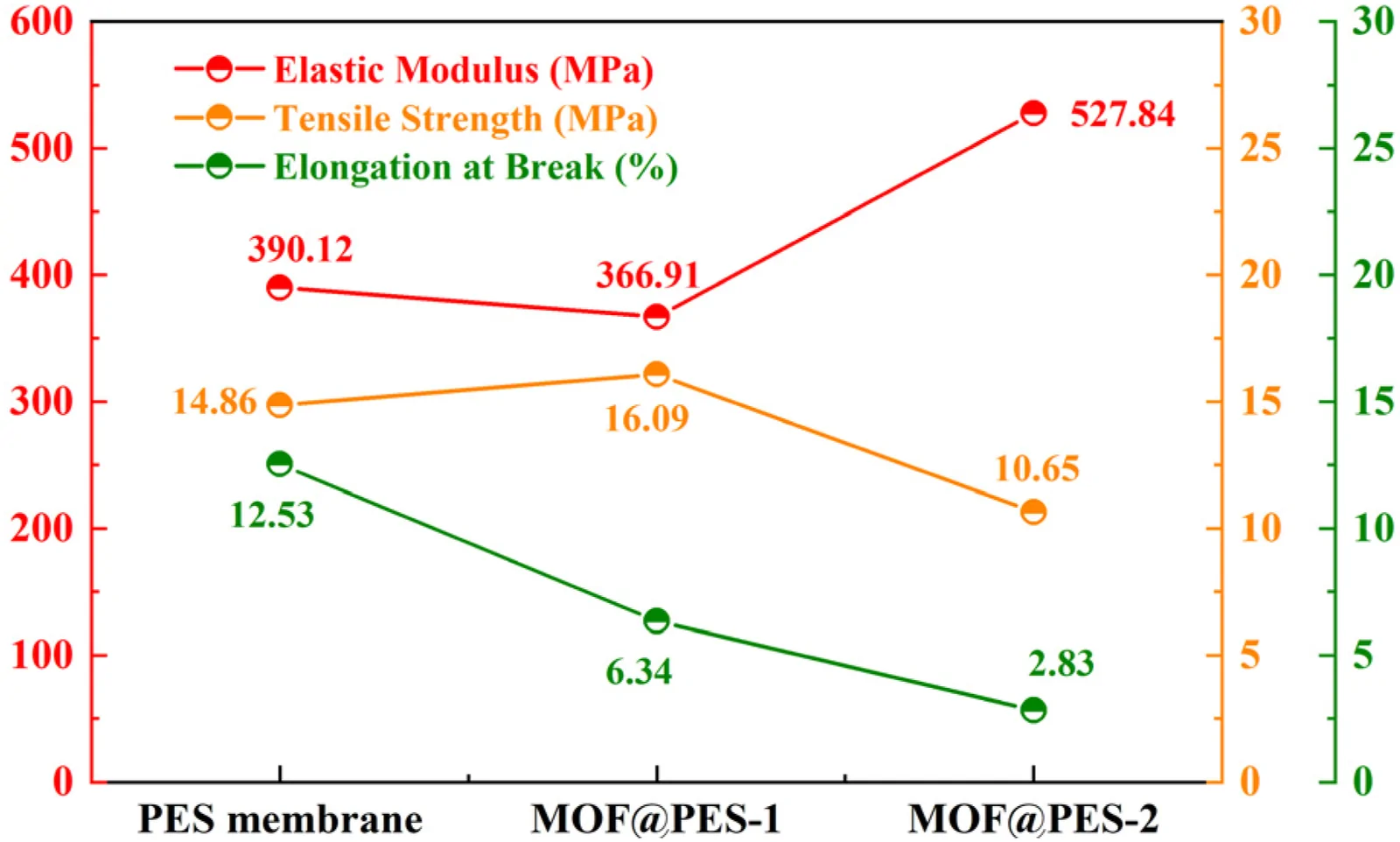
Mechanical performance data of the obtained membranes.

In addition, the water contact angles of the membranes were also measured by the sessile drop method, with three independent measurements taken at different locations for each membrane. The results are reported as mean ± standard deviation (*n* = 3). As displayed in Fig. S15–17, the neat PES membrane exhibited a contact angle of 101.77° ± 0.40°, indicating a relatively hydrophobic surface. Upon MOF-808 incorporation, the contact angle decreased to 70.93° ± 0.98° for MOF@PES-1 and further to 65.96° ± 0.36° for MOF@PES-2. This pronounced reduction confirms that the hydrophilic MOF-808 particles enhance the membrane hydrophilicity, which facilitates the access of aqueous MO species to the adsorption sites, thereby promoting the adsorption process.

### MO removal properties of membranes

Following the fabrication of the MOF-loaded MMMs, their MO removal performance was evaluated using a vacuum filtration setup (Fig. S18). In a typical test, a given volume of MO solution (30 mg L^−1^) was filtered through the membrane under a vacuum pressure of approximately 0.09 MPa, and the permeate was collected within a few minutes. The removal efficiency, calculated based on eqn (1), was defined as the percentage decrease in MO concentration after a single filtration cycle. As depicted in Fig. 7a, the pristine PES membrane exhibits a low removal efficiency of only 19.12%, confirming that the bare polymer matrix contributes little to MO adsorption. In contrast, the incorporation of MOF-808 significantly enhances performance, with removal efficiencies reaching 92.55% for MOF@PES-1 and 98.85% for MOF@PES-2. This substantial improvement indicates that the embedded MOF-808 particles serve as the primary active sites for MO adsorption. Notably, a 66.7% increase in MOF loading from 0.45 g to 0.75 g yielded only a 6.3 percentage-point gain (from 92.55% to 98.85%), indicating a clear diminishing return in performance with increasing filler content. Moreover, MOF@PES-2 already exhibited a marked decrease in elongation at break compared to MOF@PES-1, reflecting increased brittleness; further loading would inevitably promote filler agglomeration and stress concentration within the polymer matrix,^36^ rendering the MMMs prone to cracking during blade casting and thus compromising their practical applicability. Considering the trade-off among adsorption performance and mechanical durability (acceptable integrity), MOF@PES-2 was identified as the optimal formulation, offering competitive removal efficiency while maintaining adequate membrane integrity and fabricability for practical water treatment applications.

**Fig. 7:**
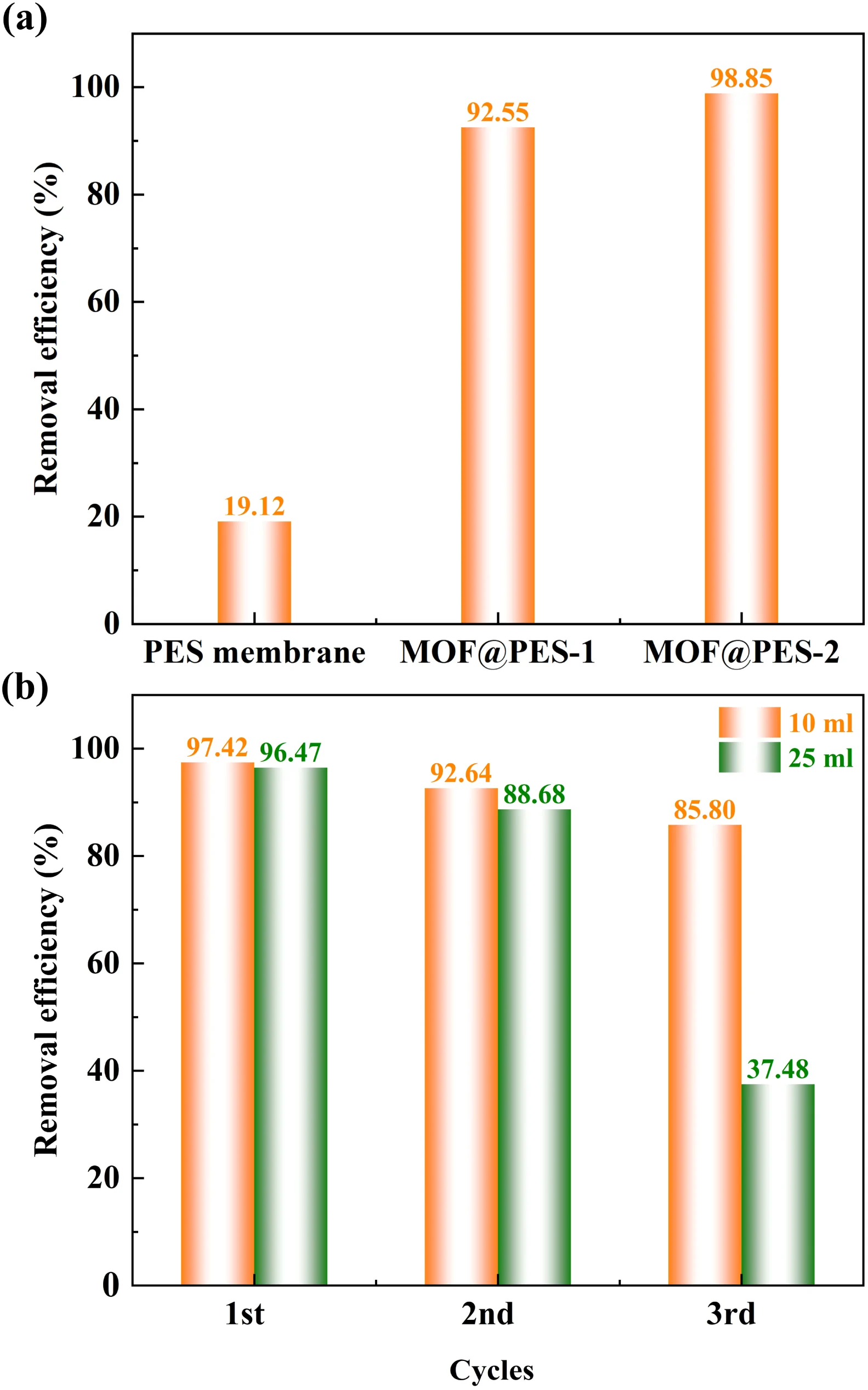
(a) Removal efficiency of MO by the three membranes (*C*_0_ = 30 mg L^−1^, *V* = 10 mL). (b) Removal efficiency of MOF@PES-2 toward MO solution over three cycles (*C*_0_ = 30 mg L^−1^).

The regeneration and reusability of the prepared MMMs were evaluated over three consecutive adsorption–desorption cycles at initial MO volumes of 10 mL and 25 mL, during which industrial ethanol was used as an eluent; the data for each cycle represent the average of three independent experiments. As shown in Fig. 7b and Tables S8–S9, both volume groups exhibited mean MO removal efficiencies above 96% in the first cycle (97.42% for 10 mL and 96.47% for 25 mL), with standard deviations of 1.48% and 2.46%, respectively, indicating that the first-cycle MO removal performance of MOF@PES-2 is highly reproducible. In the second cycle, the mean removal efficiencies were 92.64% (10 mL) and 88.68% (25 mL), with standard deviations of 3.63% and 9.03%, respectively. Although the efficiencies decreased compared with the first cycle, they still remained around 90%, indicating that most of the MOF@PES-2 performance can be regenerated with the aid of industrial ethanol. Notably, the 25 mL group exhibited a relatively larger standard deviation, suggesting greater data fluctuation. However, the removal performance of the membrane showed considerable variation in the third cycle, with notable differences between the two volume groups. For the 10 mL group, the mean removal efficiency decreased to 85.80%, with a standard deviation of 5.15%, which was 6.84% lower than that in the second cycle, indicating that most of the membrane performance could still be largely maintained. In contrast, for the 25 mL group, the mean removal efficiency dropped sharply to 37.48%, with the highest value being only 75.46%; moreover, the data fluctuated considerably, with a standard deviation of 33.10%, suggesting that the membrane material may have undergone detachment of MOF-808 particles or structural degradation during repeated regeneration.

To verify this interpretation, PXRD analysis was performed at first. As shown in Fig. S19, all the membrane material still exhibited diffraction peak intensities similar to the pristine membrane after cycling tests, indicating that most of the MOF-808 remained intact within the membrane material. However, SEM characterization of the regenerated membrane revealed distinct morphological differences between the two groups. As shown in Fig. S20–S21, the 10 mL group exhibited well-preserved surface and interfacial morphologies with negligible changes, whereas the 25 mL group showed pronounced cracks and structural degradation in both surface and cross-sectional morphologies, consistent with its sharp decline in removal efficiency. EDS analysis further revealed a slight decrease in Zr content after the tests, from 10.24% to approximately 9.00% and 9.23% for the 10 mL and 25 mL groups, respectively, indicating local detachment of MOF particles (Fig. S22–S24). For the 10 mL group, ICP-OES analysis showed no detectable Zr in the filtrate, indicating no detectable leaching of Zr ions under this condition (Table S10). Therefore, for the 10 mL group, the decrease in Zr content is more likely attributable to the industrial ethanol-assisted recovery process rather than to the filtration process. Overall, these results indicate that although the membrane exhibits good stability under moderate conditions, its long-term structural robustness during repeated regeneration at higher loadings still requires further optimization.

In addition to the structural changes of the membrane material, its mechanical properties also changed considerably. Mechanical testing (Tables S11–12) revealed a softening rather than embrittlement behaviour after three cycles. For the 10 mL and 25 mL groups, the mean value of tensile strength decreased from 10.65 to 3.09 and 4.50 MPa, the mean value of elongation at break increased from 2.83% to 5.76% and 5.30%, and the mean value of elastic modulus decreased from 527.84 to 86.12 and 138.09 MPa, respectively. This behaviour might be attributed to water-induced plasticization, which increases polymer chain mobility and reduces stiffness.^36^ In addition, the observation that the 25 mL group exhibited a higher residual tensile strength than the 10 mL group may be partially explained by the sampling locations for mechanical testing, which potentially avoided the cracked regions. Despite these changes, all cycled membranes remained handleable, and their residual tensile strength (3.09–4.50 MPa) far exceeds the typical operational stress in low-pressure dead-end filtration (<0.1–0.2 MPa),^37,38^ indicating that the membranes still possess sufficient mechanical strength for practical operation.

Finally, considering the ubiquity of inorganic salts (*e.g.*, NaCl) in practical dye wastewater, the tolerance of MOF@PES-2 to inorganic salts was evaluated using NaCl-containing MO solutions (MO: 30 mg L^−1^, 10 mL). As shown in Fig. 8, at a NaCl concentration of 30 mg L^−1^ (equivalent to the MO mass concentration), the membrane maintained a high removal efficiency exceeding 95%; even when the NaCl concentration was increased tenfold to 300 mg L^−1^, MOF@PES-2 still achieved a removal efficiency of 89.07%, corresponding to a moderate decrease from 98.85% in the absence of NaCl. To investigate the cause of this decline, the membrane after filtration with a 300 mg L^−1^ NaCl solution was first characterized by XRD, SEM, and EDS elemental mapping, as shown in Fig. S25–S27. No obvious changes were observed in the XRD patterns or SEM images. EDS analysis further showed that the Zr content did not change significantly, with only a slight fluctuation from 10.24% to 10.32%. These results suggest that the reduced removal efficiency was not primarily caused by significant changes in the crystalline structure, morphology, or Zr content of the membrane. Considering that the MO removal performance of MOF-808 is strongly influenced by its surface charge, the decline in removal efficiency was more likely related to changes in surface charge. Therefore, the zeta potentials of MOF-808 in NaCl solutions with concentrations of 0, 30, and 300 mg L^−1^ were measured. As shown in Fig. 8 and Table S13, the zeta potential decreased from 32.00 mV at 0 mg L^−1^ to 30.03 and 26.77 mV at 30 and 300 mg L^−1^, respectively. This variation in zeta potential was consistent with the decline in removal efficiency with increasing NaCl concentration. To further verify this hypothesis, the removal efficiency of the membrane was evaluated using a MO solution (30 mg L^−1^, 10 mL) containing 3000 mg L^−1^ NaCl. As expected, the zeta potential dropped sharply to 14.77 mV, and the removal efficiency of MOF@PES-2 for MO simultaneously decreased to 46.54%. These results strongly support the hypothesis that the decrease in zeta potential weakened the electrostatic attraction between MOF-808 and the anionic MO molecules, thereby leading to the decline in removal efficiency.

**Fig. 8:**
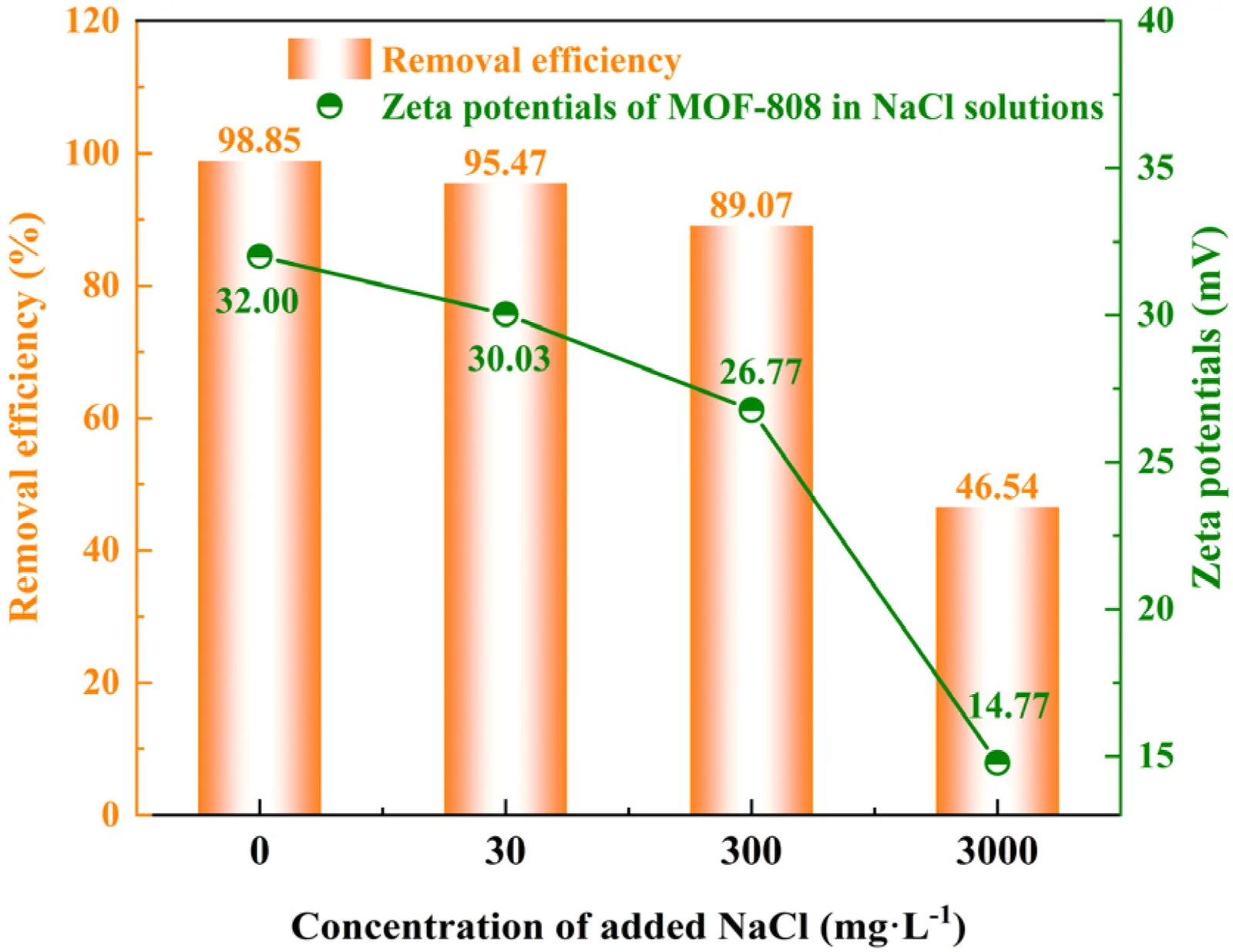
Dual *y*-axis plot showing the removal efficiency of MOF@PES-2 toward a 30 mg L^−1^ MO solution containing NaCl (left *y*-axis) and the zeta potential of MOF-808 in NaCl solutions of different concentrations (right *y*-axis).

In addition, changes in the mechanical properties of the membrane after filtration of an MO solution (30 mg L^−1^, 10 mL) containing 300 mg L^−1^ NaCl were also evaluated. As shown in Table S14, the mean value of elastic modulus recovered to 217.77 MPa, while the average of elongation at break decreased to 3.09%. This suggests that salt ions may induce partial crosslinking or dehydration of the polymer chains, thereby restoring some rigidity. Nevertheless, the membrane still achieved a removal efficiency of 89.07% while preserving its morphological integrity and mechanical robustness. These results indicate that the fabricated membrane maintains robust MO removal performance in NaCl-containing aqueous environments.

### Comparison and outlook

As shown in Table S15, the MOF-808@PES-2 membrane achieved an initial methyl orange (MO) removal efficiency exceeding 96% (>97% within three cycles in the 10 mL system and >96% within two cycles in the 25 mL system), which is comparable to several high-performance MOF-based membranes (>95%). However, its reusability remains insufficient: only 1∼3 cycles were examined, and the MO removal retention decreased to 85.80–88.68% after 2∼3 cycles, which still falls short of literature values. Notably, the membrane exhibited some salinity tolerance in NaCl-containing systems: under an equal mass concentration of NaCl, MO removal remained >95%; even at a 10-fold higher mass concentration of NaCl, the removal was still >89%. Considering that stability tests in the literature were mostly conducted in pure water or low-salinity systems, these results suggest that the membrane shows potential for application under high-salinity conditions. Future work will focus on conducting longer-term cyclic and continuous cross-flow tests and further improving long-term operational stability by enhancing MOF–PES interfacial adhesion, optimizing regeneration strategies, and regulating MOF loading and pore structure.

## Conclusions

In summary, our study reveals that the superior MO adsorption capability of MOF-808 can be attributed to surface charge matching between MOF-808 and MO molecules. Building upon this, MOF-808-loaded mixed matrix membranes (MMMs) were successfully fabricated and systematically evaluated for the adsorptive removal of MO from aqueous solutions. Experimental results demonstrated that, compared to the pristine PES membrane, the MOF-loaded MMMs exhibited significantly enhanced MO removal performance. Notably, MOF@PES-2 exhibited the best overall performance, achieving a maximum MO removal efficiency of 98% for a 30 mg L^−1^ MO solution (10 mL), and maintaining an efficiency exceeding 85% over three consecutive filtration cycles (10 mL per cycle). Moreover, even in the presence of NaCl at a mass concentration equal to that of MO, the membrane retained an impressive MO removal efficiency exceeding 95%. Taken together, these findings highlight the great promise of the MOF-808-incorporated MMMs as highly efficient and practical candidates for dye wastewater remediation. However, the mechanical data showed considerable sample-to-sample heterogeneity, especially at higher MOF loadings, suggesting that the current fabrication method has limitations and should be further optimized rather than simply increasing the MOF loading. Future work should focus on overcoming the limitations of the current fabrication method, improving membrane homogeneity and mechanical robustness, establishing a standard filtration system for water flux/permeance, antifouling measurements, and evaluating long-term performance under realistic saline wastewater conditions.

## Author contributions

Xiao-Jing Hu: experimental design and protocol formulation, experimental operation, data curation, methodology, writing – original draft. Qi-Qiang Li: experimental operation, data curation, software. Li-Jie Lin: data curation, visualization. Xiao-Hui Huang: funding acquisition, supervision, writing – review & editing. Shao-Ming Ying: formal analysis, writing – review & editing.

## Conflicts of interest

There are no conflicts to declare.

## Supplementary Material

RA-OLF-D6RA07189A-s001

## Data Availability

All data supporting the findings of this study are available within the article and its supplementary information (SI). Supplementary information is available. See DOI: https://doi.org/10.1039/d6ra07189a.
