# Supplementary material for: Highly efficient MOF-808/polyethersulfone mixed matrix membranes for methyl orange filtration
Source: RSC Adv. 2026 Oct 19;16(48):64270–8. doi: 10.1039/d6ra07189a (PMC13678692; doi:10.1039/d6ra07189a)
Supplement: Supplementary file 1 [file RA-016-D6RA07189A-s001.pdf]

Supplementary Information (SI) for RSC Advances.
This journal is © The Royal Society of Chemistry

Highly Efficient MOF-808/Polyethersulfone Mixed Matrix Membranes for Methyl Orange Filtration

Xiao-Jing Hu,[a] Qi-Qiang Li,[a] Li-Jie Lin,[a] Xiao-Hui Huang*[a] and Shao-Ming Ying*[a]

1. Materials and Instruments
2. Synthesis of MOF-808 samples
3. Characterization of MOF-808
4. Methyl orange (MO) adsorption experiments of MOF-808
5. XPS of MOF-808 before and after MO adsorption
6. Methylene blue adsorption experiment of MOF-808
7. Photos of prepared membranes
8. SEM image and EDS mapping of prepared membranes
9. ICP-OES analysis of membranes
10. Mechanical performance data of the obtained membranes
11. Water contact angles of the membranes
12. The experimental setup for evaluating the MO removal performance
13. Cycling test results of MOF@PES-2

14. Characterization of MOF@PES-2 after cyclic experiments

15. Characterization of MOF@PES-2 after filtration of NaCl-containing MO solutions

16. Comparison with recent literature for methyl orange removal

17. References

1. Materials and Instruments

All chemical reagents and solvents were purchased from commercial sources and used as received without further purification. Powder X-ray diffraction (PXRD) patterns were recorded on a Rigaku SmartLab diffractometer. Nitrogen (N_2) adsorption-desorption measurements were performed using a Micromeritics ASAP 2460 analyzer. Thermogravimetric analysis (TGA) was performed using a NETZSCH (Germany) thermal analyzer under a nitrogen atmosphere from 30 to 1000 °C at a heating rate of 10 °C min^{-1}. The morphology of the MOF-808 nanoparticles was characterized by scanning electron microscopy (SEM, Zeiss Sigma 300) operating at an accelerating voltage of 10 kV. Zeta potential and particle size distribution analyses of MOF-808 were conducted on a Malvern Zetasizer Nano ZS90. X-ray photoelectron spectroscopy (XPS) measurements were performed using a Thermo Scientific K-Alpha spectrometer (Thermo Fisher Scientific, USA). Inductively coupled plasma optical emission spectrometry (ICP-OES) measurements were performed using an Agilent 5800 ICP-OES spectrometer (Agilent Technologies, USA). The concentrations of MO/MB were determined using a UV-vis spectrophotometer (UV-8000S, Shanghai Metash Instruments Co., Ltd.). Static water contact angles (WCAs) of the membranes were measured using a contact angle goniometer (SDC-350KS, Kunshan Shengding, China). Mercury intrusion porosimetry (MIP) measurements were performed using a Micromeritics AutoPore IV 9500 porosimeter (Micromeritics, USA). FTIR spectra were collected using a Thermo Scientific Nicolet iS20 spectrometer (Thermo Fisher Scientific, USA) equipped with an attenuated total reflectance (ATR) accessory.

2. Synthesis of MOF-808 samples

The white nanoparticles of MOF-808 were synthesized according to a previously reported method with minor modifications. [1, 2] Typically, the ligand H_3BDC (105 mg) was dissolved in N, N-Dimethylmethanamide (DMF, 22.5 mL) via ultrasonication in a Teflon-lined stainless-steel autoclave. Subsequently, $ZrOCl_2{\cdot}8H_2O$ (385 mg) and formic acid (12.5 ml) were added to the solution, and the mixture was further sonicated for 15 min to ensure complete dissolution. The autoclave was then sealed and heated at 130 °C for 48 h. After cooling to room temperature, the white precipitate was collected by centrifugation, washed thoroughly with fresh DMF, and sequentially soaked in DMF and acetone/ethanol for 3 days each. Finally, the solvent-exchanged MOF-808 was dried under vacuum at room temperature.

3. Characterization of MOF-808

3.1 PXRD patterns of MOF-808

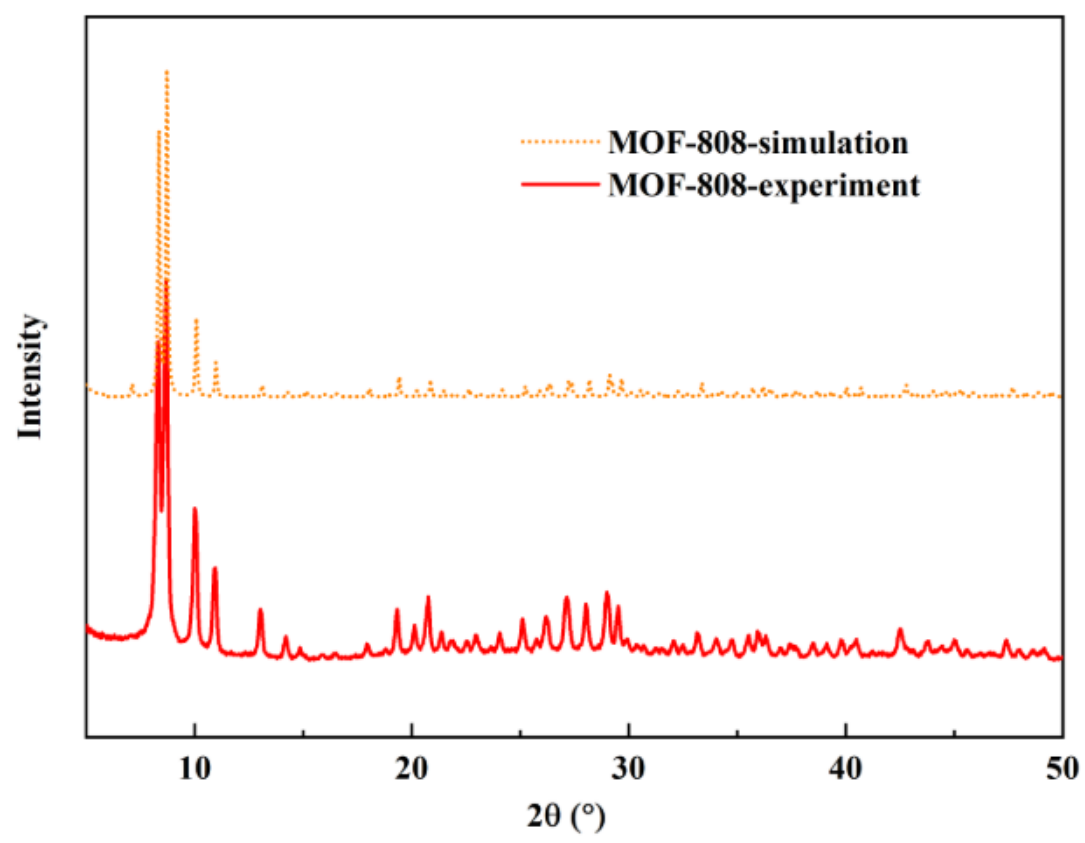

Fig. S1 PXRD patterns of the as-synthesized MOF-808.

3.2 N_2 adsorption isotherms of MOF-808

Prior to N_2 sorption measurements, the as-synthesized MOF-808 samples were washed several times with DMF and immersed in fresh DMF for 3 days, with the solvent refreshed three times. To remove the framework-confined DMF molecules, the samples were

subsequently immersed in ethanol for another 3 days, during which the solvent was also replaced three times. The samples were then isolated and activated at 100 °C under vacuum for 12 h.

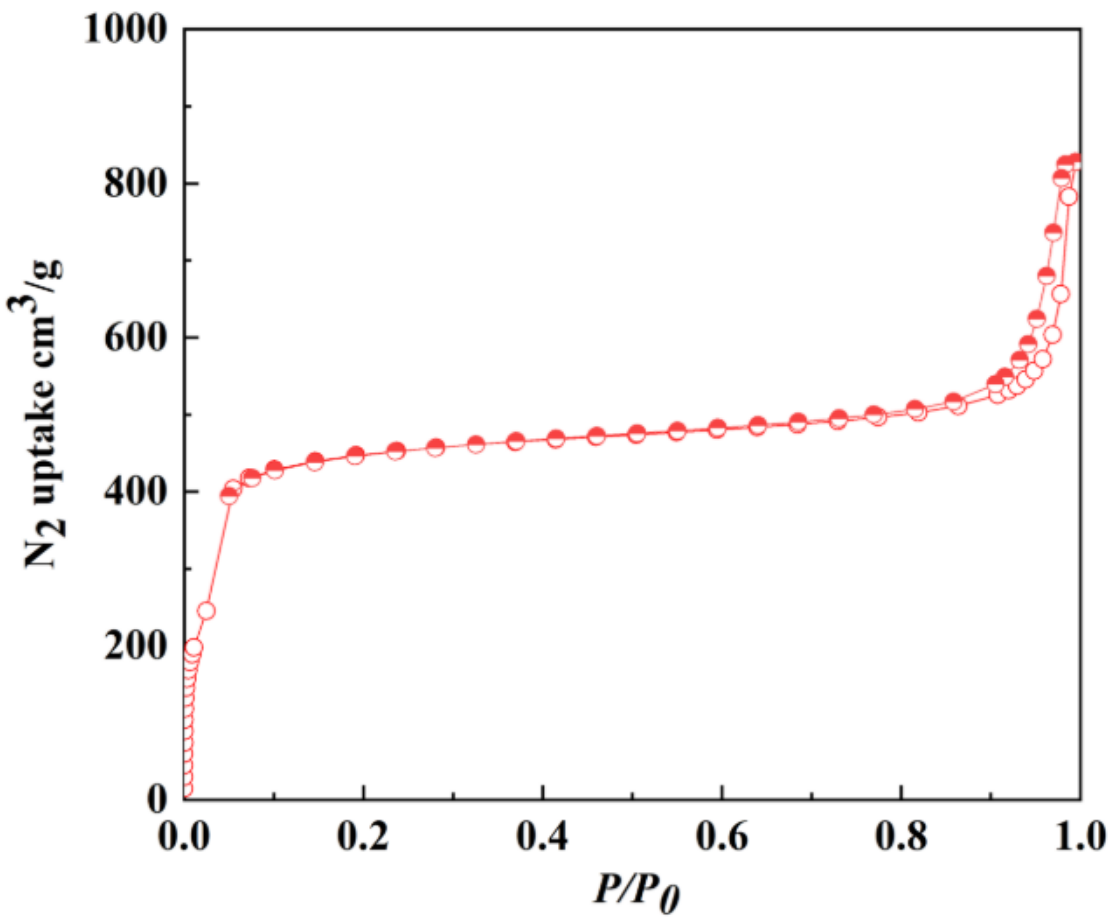

Fig. S2 N_2 adsorption/desorption isotherms (77 K) of the as-synthesized MOF-808.

3.3 TGA curve of pristine MOF-808

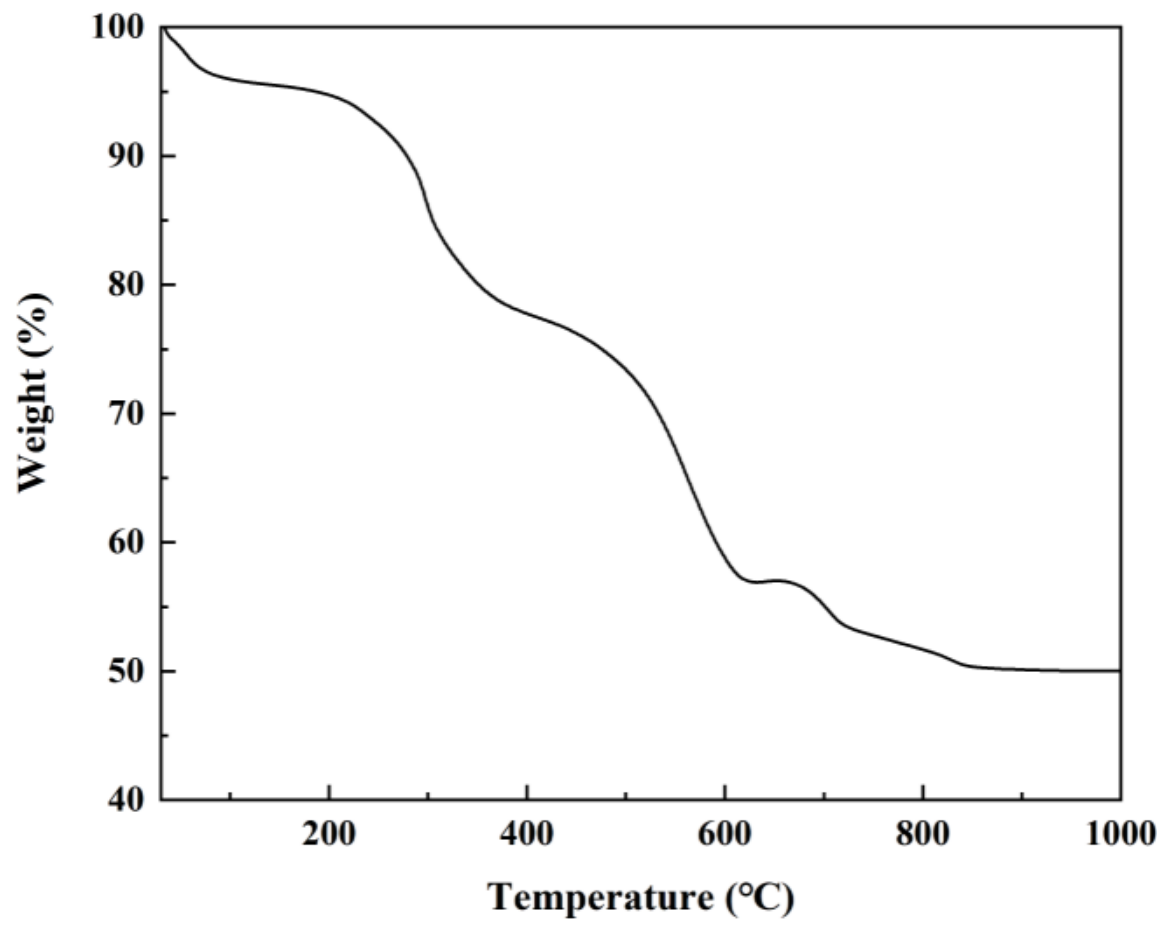

Fig. S3 TGA curve of the as-synthesized MOF-808 under N₂ atmosphere.

3.4 SEM images of MOF-808

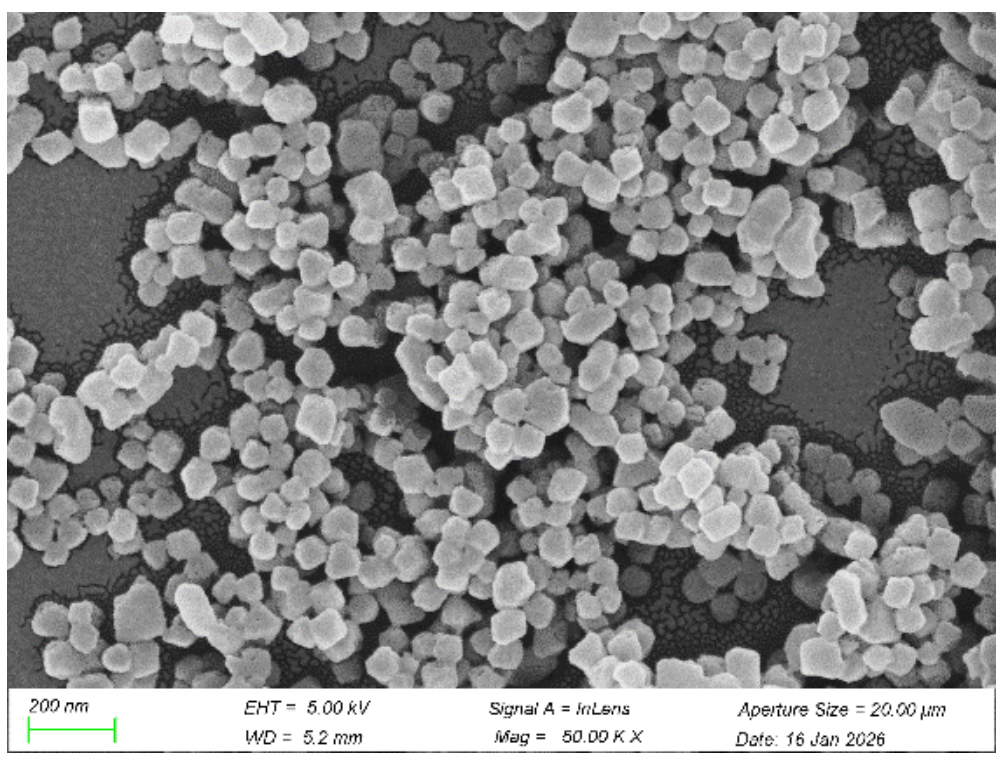

Fig. S4 SEM image of MOF-808 nanoparticles.

3.5 Size distribution of prepared MOF-808 particles

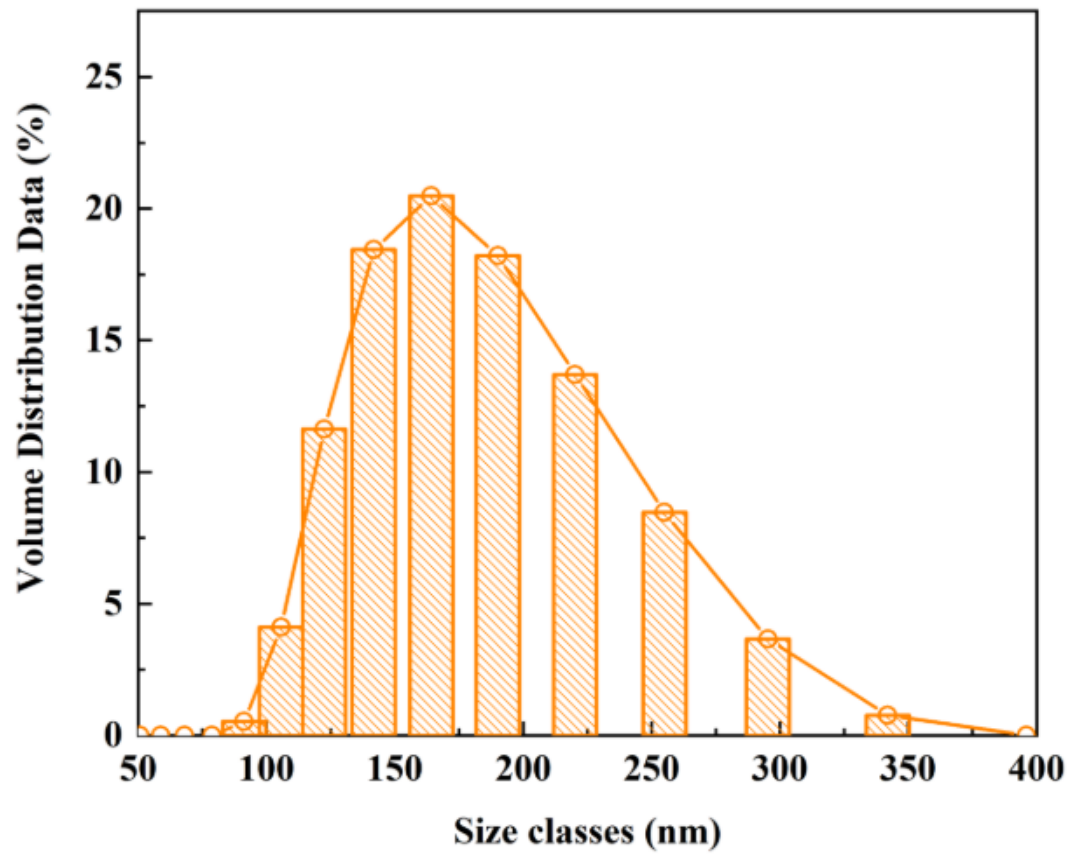

Fig. S5 Size distribution of prepared MOF-808 particles.

4. Methyl orange (MO) adsorption experiments of MOF-808

4.1 Procedures for methyl orange calibration curve

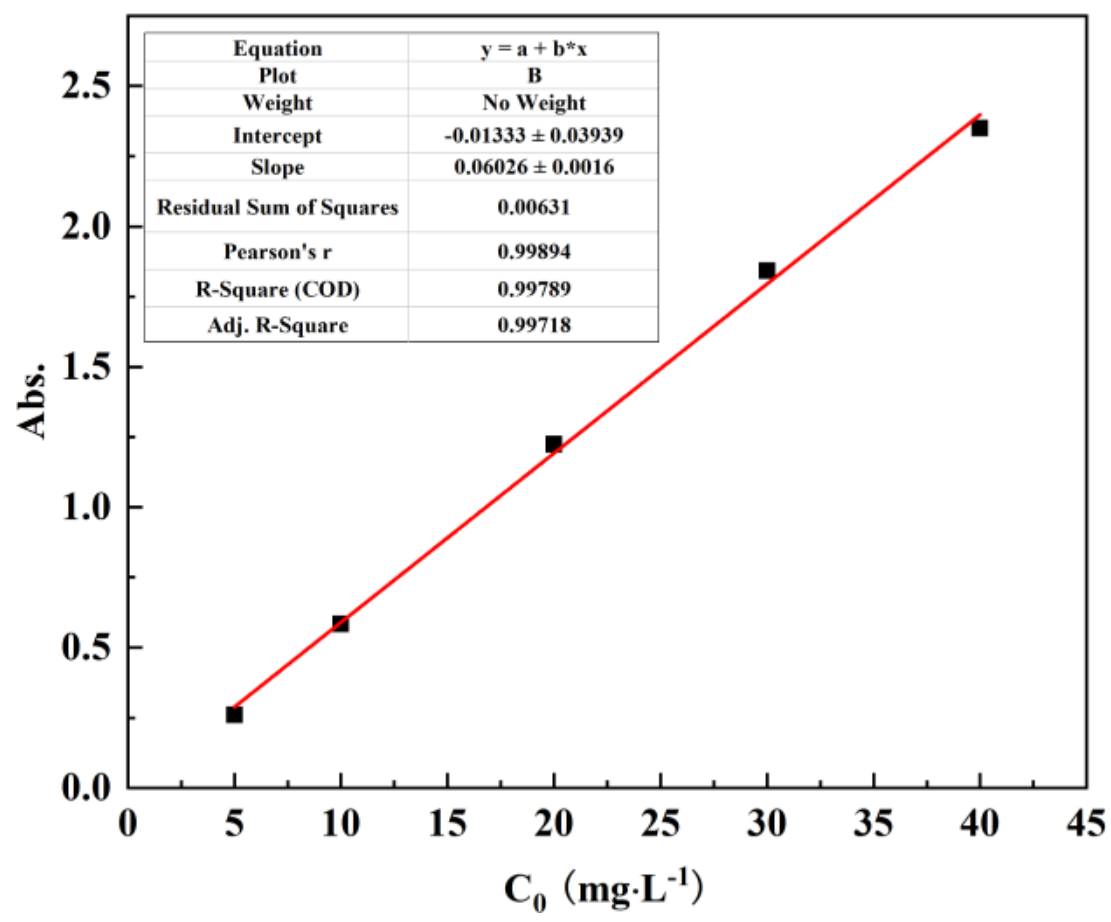

Fig. S6 Calibration curve for MO at 464 nm using standard MO solutions.

4.2 MO adsorption kinetics experiment

To evaluate the adsorption kinetics, the time-dependent MO adsorption on MOF-808 was monitored for up to 240 min. Briefly, 100 mL of a 30 mg L^{-1} MO aqueous solution in a capped conical flask was pre-shaken in a water bath (150 rpm, 25 °C) for 30 min, followed by the addition of 20 mg of activated MOF-808. At designated intervals (0, 10, 20, 40, 60, 120, and 240 min), ~2.5 mL of the supernatant was sampled and measured via a UV-vis

spectrophotometer. The MO concentration was then calculated using the absorbance values in reference to the calibration curve.

The amount of adsorbed MO was determined according to the following equations[3]:

$$Q_t=(C_o-C_t)\frac{V}{m} \qquad \text{Eq. S1}$$

where C_o (mg L^{-1}) and C_t (mg L^{-1}) are the concentrations of MO initially and at time , respectively; v (L) is the volume of the MO solution; and m (g) is the mass of the adsorbent.

To evaluate the adsorption kinetics, the experimental data were fitted using the pseudo-first-order and pseudo-second-order models.[4]

Pseudo-first-order model:

$$Q_t=Q_e{}^*(1-e^{-K^*t}) \qquad \text{Eq. S2}$$

Pseudo-second-order model:

$$\frac{t}{Q_t}=\frac{1}{K_2 Q_e^2}+\frac{t}{Q_e} \qquad \text{Eq. S3}$$

where Q_t (mg g^{-1}) and Q_e represent the adsorption capacities at time (min) and at equilibrium, respectively, and K_2 (g·min^{-1} mg^{-1}) is the rate constant of the pseudo-second-order model.

Table S1 Adsorption kinetic fitting parameters of MOF-808 for MO.

Parameters / Model	kinetics parameters		
	Q_e	K	R^2
Pseudo-first-order	125.14 mg g^{-1}	0.0611 min^{-1}	0.982
Pseudo-second-order	138.96 mg g^{-1}	0.000615 g min^{-1} mg^{-1}	0.9997

4.3 Methyl orange adsorption isotherm experiment

For adsorption isotherm measurements, 100 mL of MO aqueous solutions with different

initial concentrations (0, 20, 40, 80, 200, and 400 mg L^{-1}) in capped conical flasks were pre-shaken at 25 °C (150 rpm) for 30 min. After the addition of 10 mg of activated MOF-808, the mixtures were left undisturbed for 3 h. Subsequently, ~2.5 mL of the supernatant was collected, filtered through a syringe filter, and measured using a UV-vis spectrophotometer. The solutions with high initial configurations (200 and 400 mg L^{-1}) were diluted 10-fold before analysis. The final MO concentration was calculated based on the absorbance and the pre-established calibration curve.

The Langmuir and Freundlich isotherm models were applied to fit the experimental equilibrium adsorption data.[5]

Langmuir model:

$$Q_e = \frac{K_L * Q_{max} * C_e}{1 + K_L * C_e} \qquad \text{Eq. S4}$$

Freundlich model:

$$Q_e = K_F C_e^{1/n} \qquad \text{Eq. S5}$$

where C_e (mg L^{-1}) is the equilibrium concentration of MO; Q_e (mg g^{-1}) is the equilibrium adsorption capacity; Q_{max} (mg g^{-1}) represents the theoretical maximum adsorption capacity of MOF-808 calculated from the Langmuir model; and K_L (L mg^{-1}) and K_F are the Langmuir and Freundlich isotherm constants, respectively.

Table S2 Isotherm model parameters for MO adsorption on MOF-808.

Parameters / Models	Adsorption isothermal parameters			
	K_L	K_F	Q_{max}	R^2
Freundlich		109.35		0.815
Langmuir	0.0356 L mg^{-1}		646.98 mg g^{-1}	0.968

4.4 Comparison of various Zr-MOFs for MO removal

Table S3 Comparison of MO adsorption capacities of representative Zr-MOFs (2023～2026).

Adsorbent	Q_{max}(mg g^{-1})	Year	Reference
MOF-525	854.30	2024	6
MIL-140A	163.6	2024	7
UiO-66 (mixed linker)	202.82	2024	8
FSUNi(10)	200.803	2023	9
Zr-NDC MOF	90	2026	10
MOF-808	646.98	This work	/

5. XPS of MOF-808 before and after MO adsorption

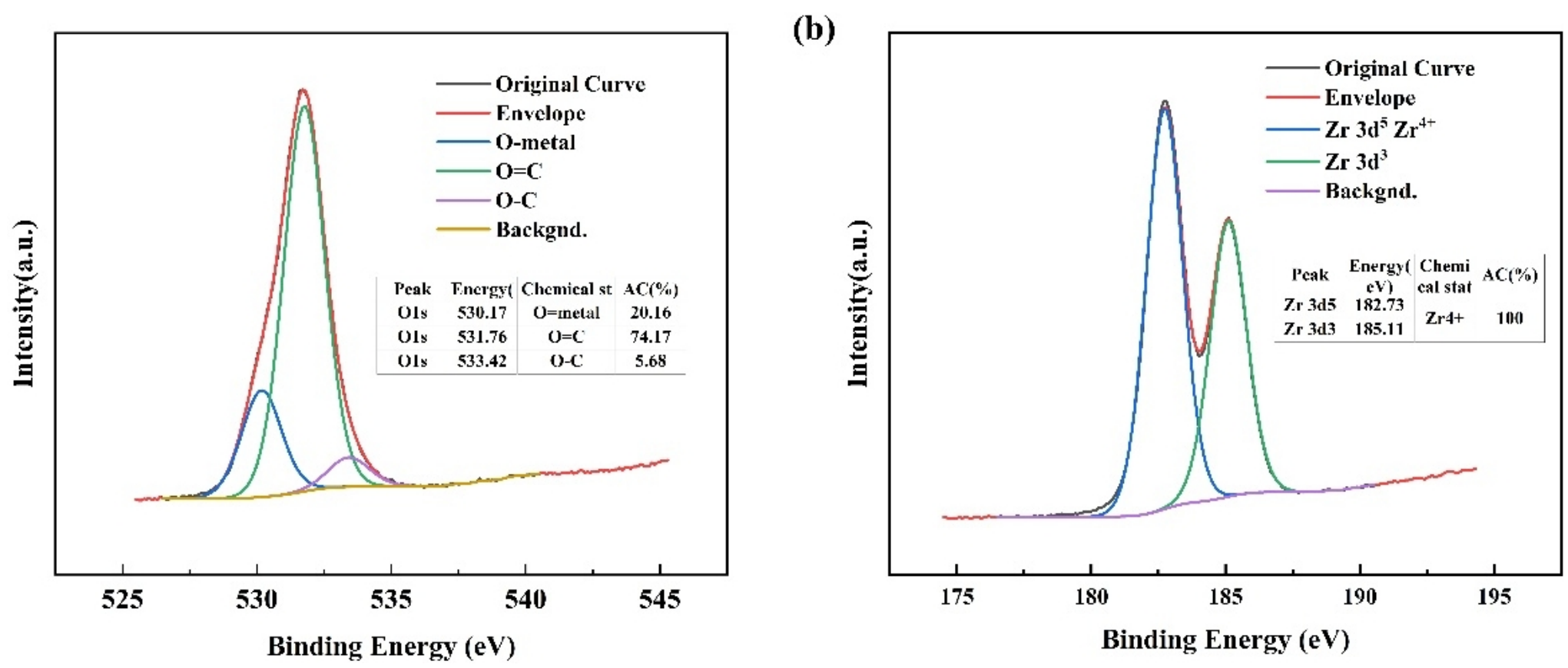

Fig. S7 XPS spectra of MOF-808 before adsorption.

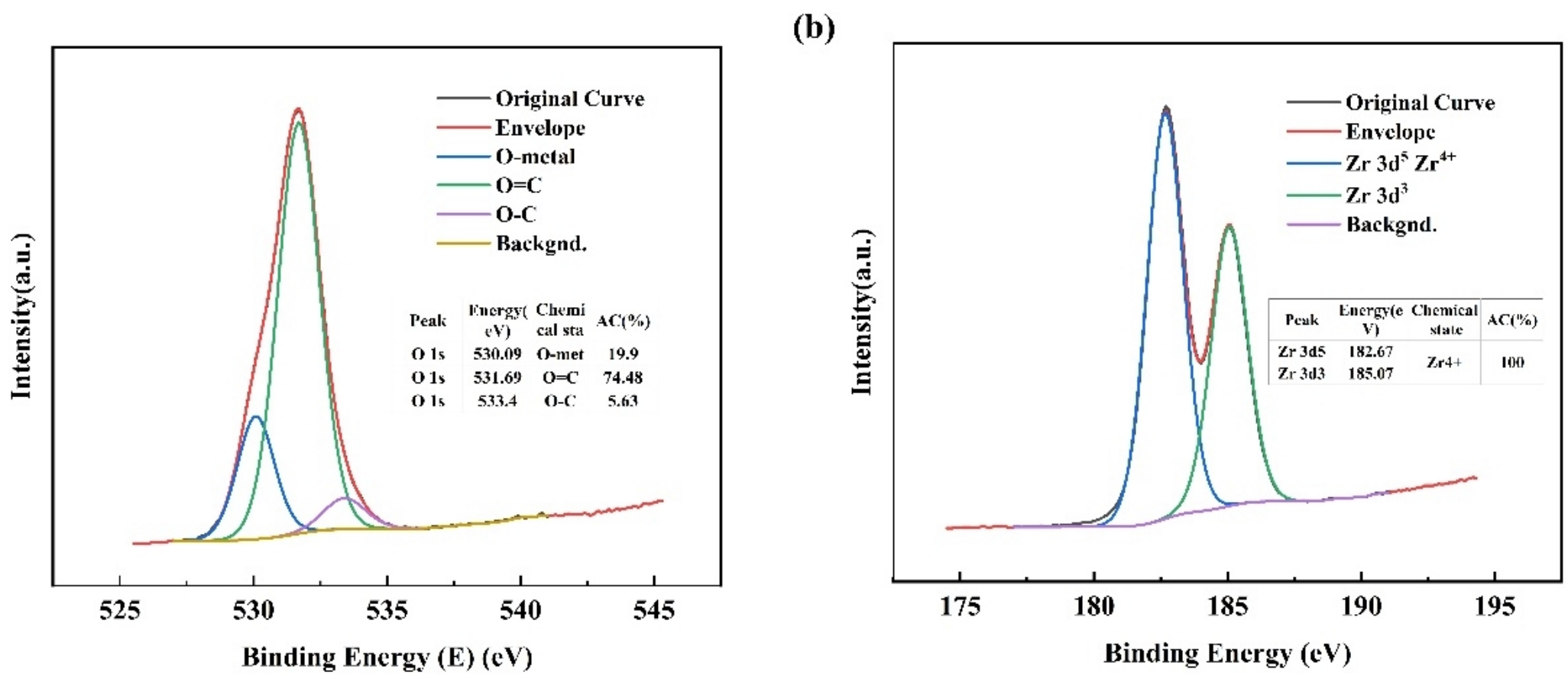

Fig. S8 XPS spectra of MOF-808 after adsorption.

6. Methylene blue (MB) adsorption experiment of MOF-808

Typically, 100 mL of an aqueous solution containing methylene blue (MB, 30 mg L^{-1}) was placed in a capped conical flask and pre-equilibrated in a shaking water bath (150 rpm, 25 °C) for 30 min. Subsequently, 20 mg of activated MOF-808 powder was added to the solution. After 240 min of adsorption, approximately 2.5 mL of the supernatant was sampled and analyzed using a UV-vis spectrophotometer.

7. Photos of prepared membranes

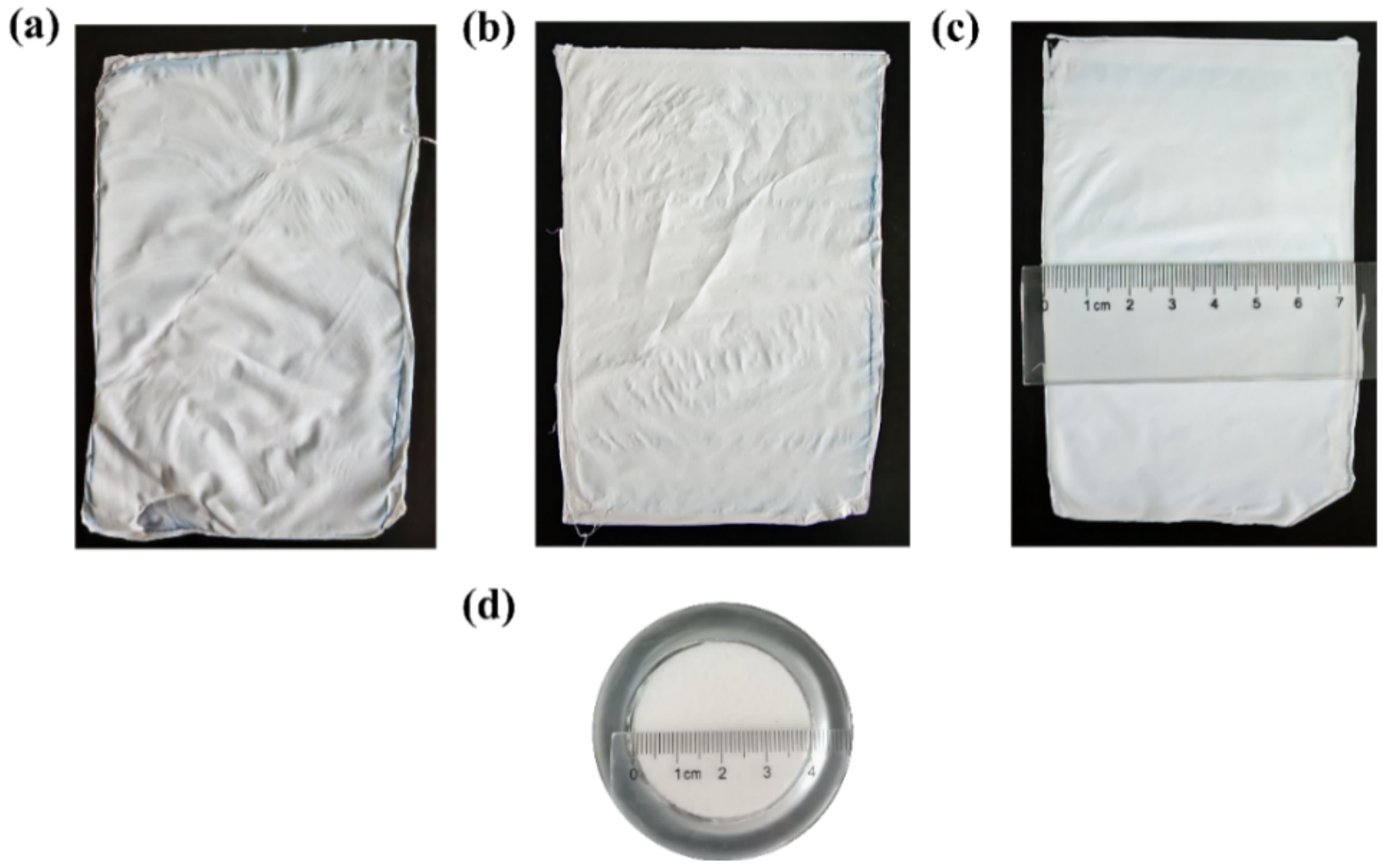

Fig. S9 (a) PES membrane; (b) MOF-PES-1; (c) MOF-PES-2; (d) filter of vacuum filtration device.

8. SEM image and EDS mapping of prepared membranes

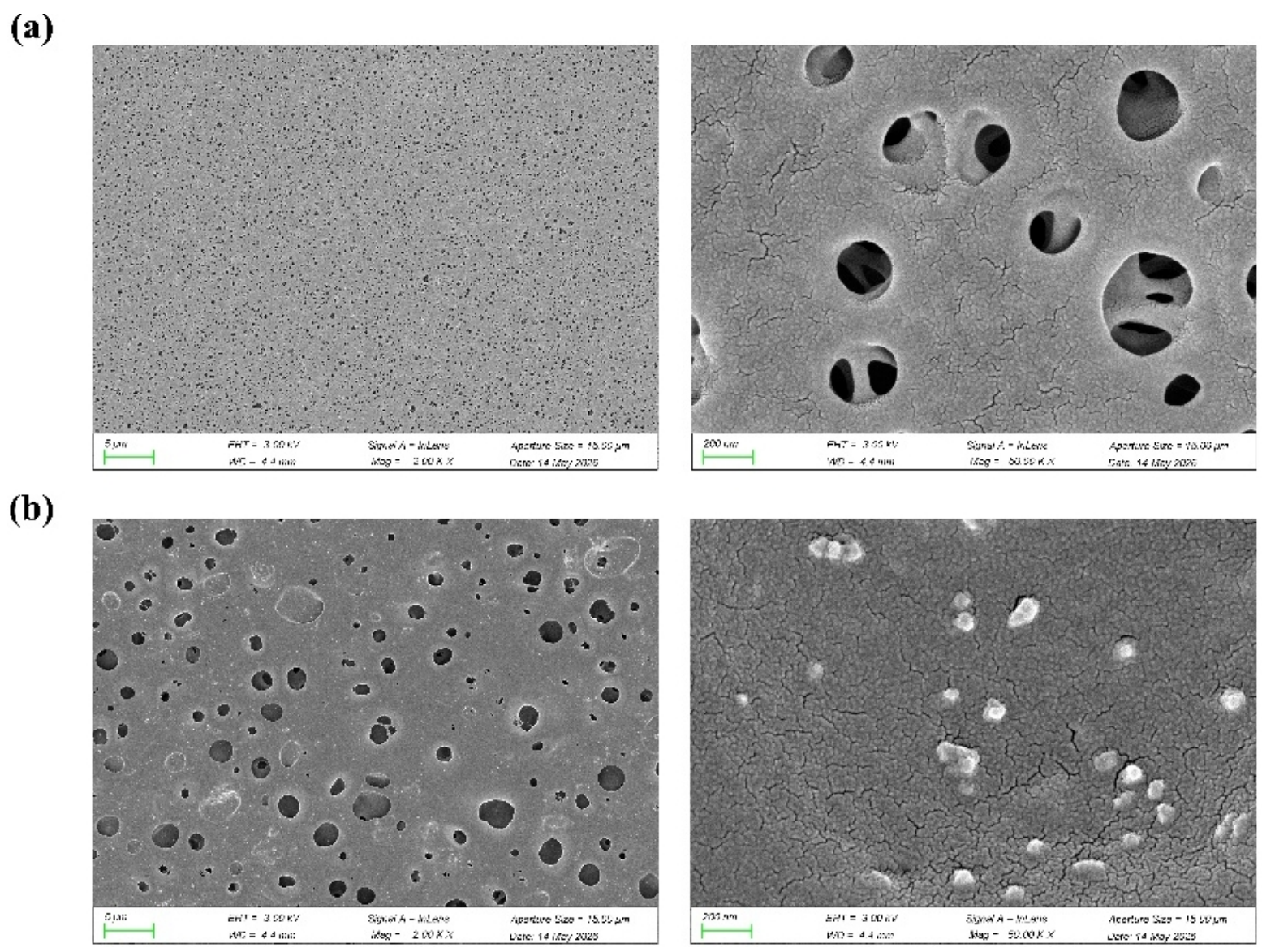

Fig. S10 Surface SEM images of the as-synthesized (a) neat PES and (b) MOF@PES-1.

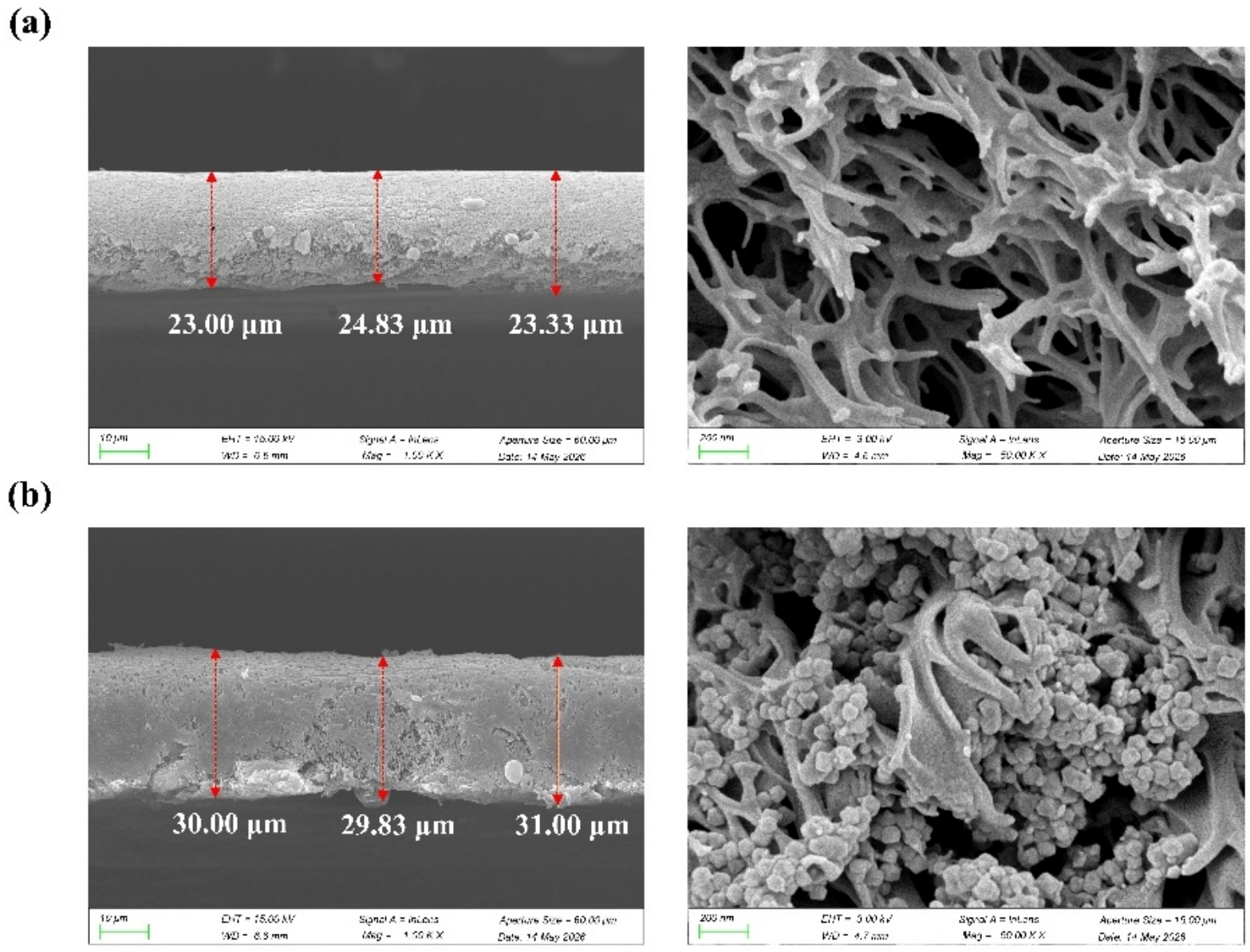

Fig. S11 Cross-sectional SEM images of the prepared (a) PES and (b) MOF@PES-1.

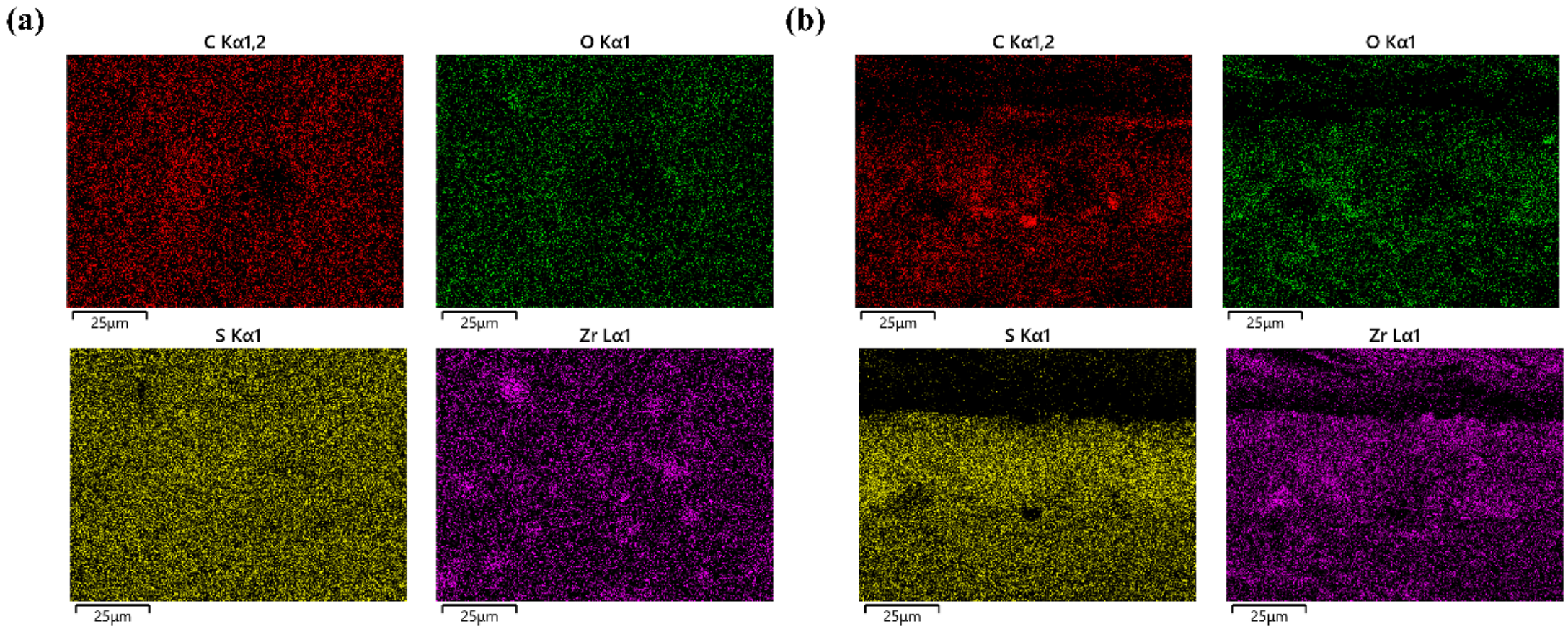

Fig. S12 (a) Surface and (b) cross-sectional EDS elemental mapping images of the MOF@PES-1.

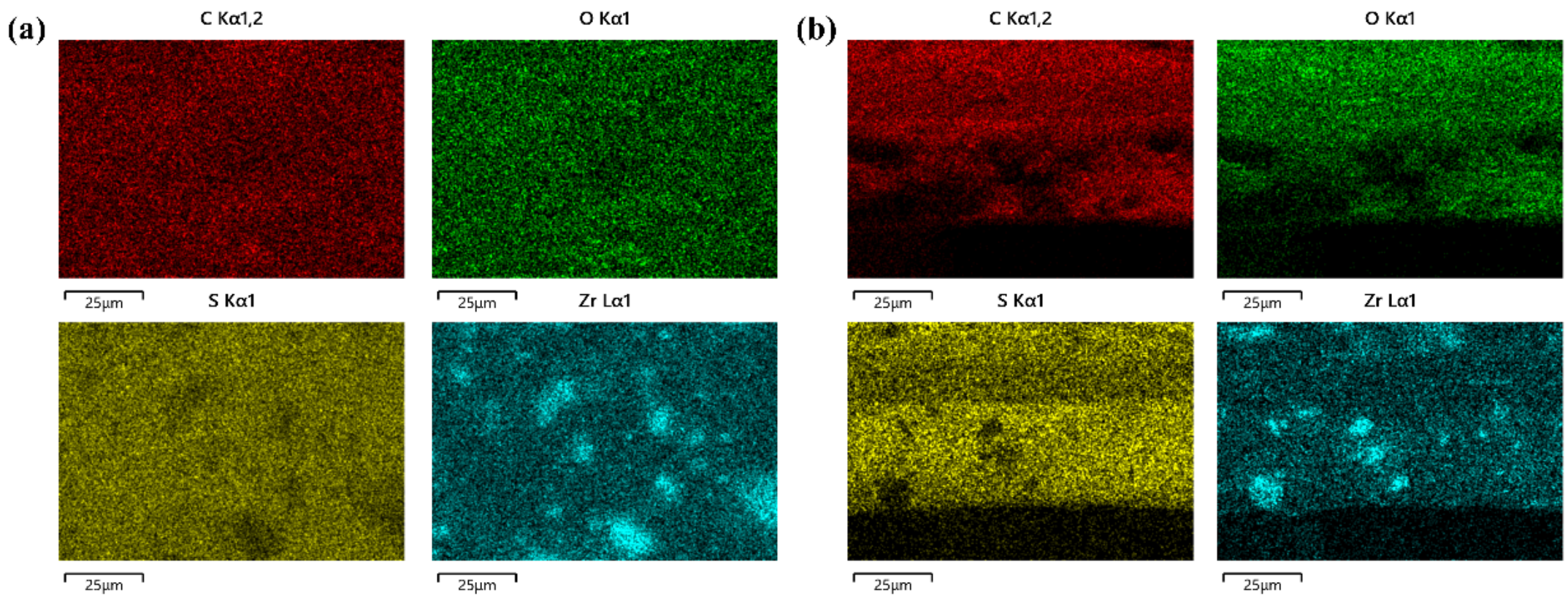

Fig. S13 (a) Surface and (b) cross-sectional EDS elemental mapping images of the MOF@PES-2.

9. ICP-OES analysis of membranes

Table S4 ICP-OES measurement of Zr content and calculated MOF-808 loading in in pristine MOF-808 and membranes.

Sample	Zr content by ICP-OES (wt%)	Calculated MOF-808 loading (wt%)	Theoretical MOF-808 loading* (wt%)
MOF-808	38.32	100	/
MOF@PES-1	5.89	15.37	23.08%
MOF@PES-2	10.84	28.29	33.33%

* Theoretical loading $= \frac{m_{MOF}}{(m_{MOF}+m_{PES})} \times 100\%$

10. Mechanical performance data of the obtained membranes

Table S5 Mechanical performance data for PES membrane

Samples number	Tensile strength/MPa	Elastic modulus/MPa	Elongation at break（%）
1	16.12	398.51	19.69
2	13.68	443.29	7.36
3	14.77	328.56	10.53
Mean	14.86	390.12	12.53
SD	1.22	57.82	6.40

Table S6 Mechanical performance data for MOF@PES-1

Samples number	Tensile strength/MPa	Elastic modulus/MPa	Elongation at break（%）
1	13.3	270.01	6.42
2	17.79	435.79	5.14
3	17.18	394.92	7.47
Mean	16.09	366.91	6.34
SD	2.44	86.37	1.17

Table S7 Mechanical performance data for MOF@PES-2

Samples number	Tensile strength/MPa	Elastic modulus/MPa	Elongation at break（%）
1	2.78	654.9	11.25
2	2.92	610.08	12.27
3	2.78	318.55	8.43
Mean	2.83	527.84	10.65
SD	0.08	182.63	1.99

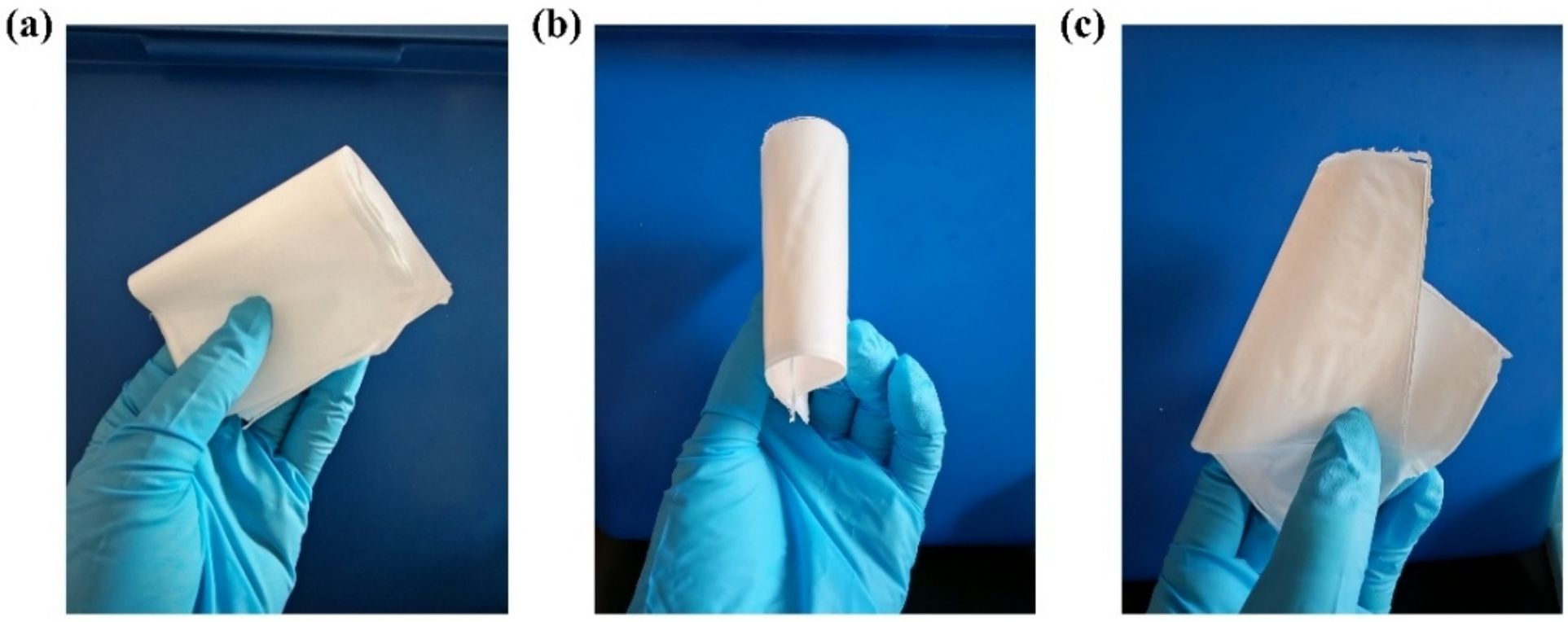

Fig. S14 Photograph of the MOF@PES-2 in a bent state.

11. Water contact angles of the membranes

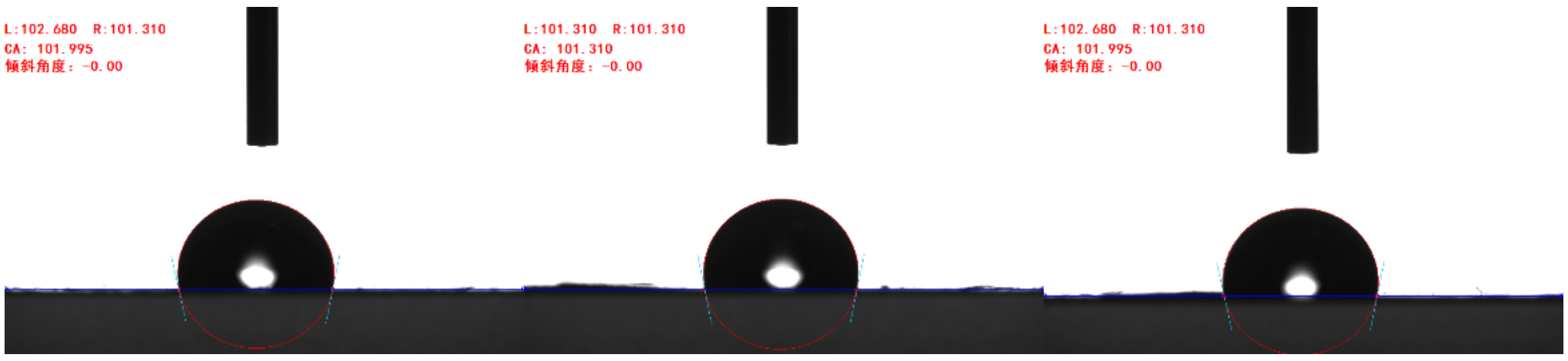

Fig. S15 Water contact angles of PES membrane.

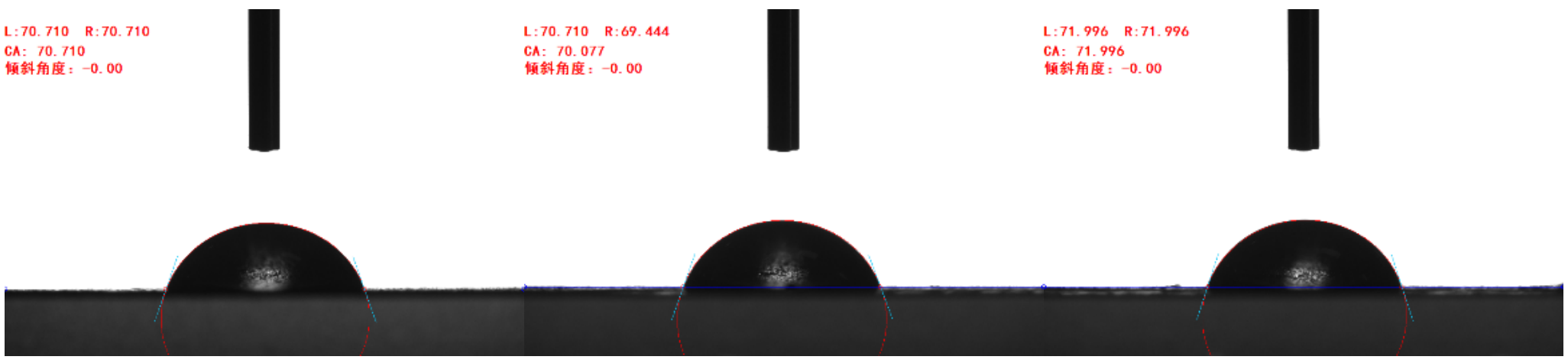

Fig. S16 Water contact angles of MOF@PES-1.

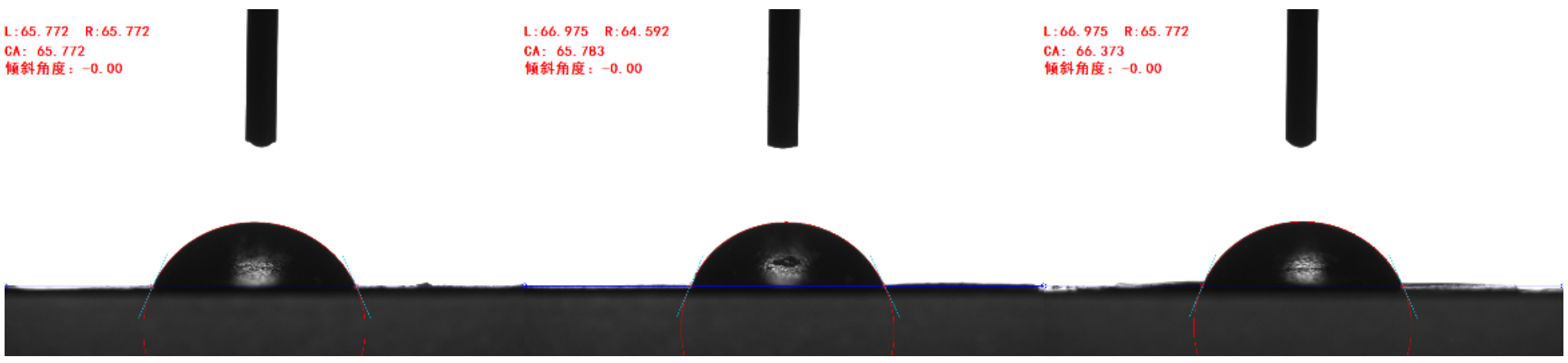

Fig. S17 Water contact angles of MOF@PES-2.

12. The experimental setup for evaluating the MO removal performance

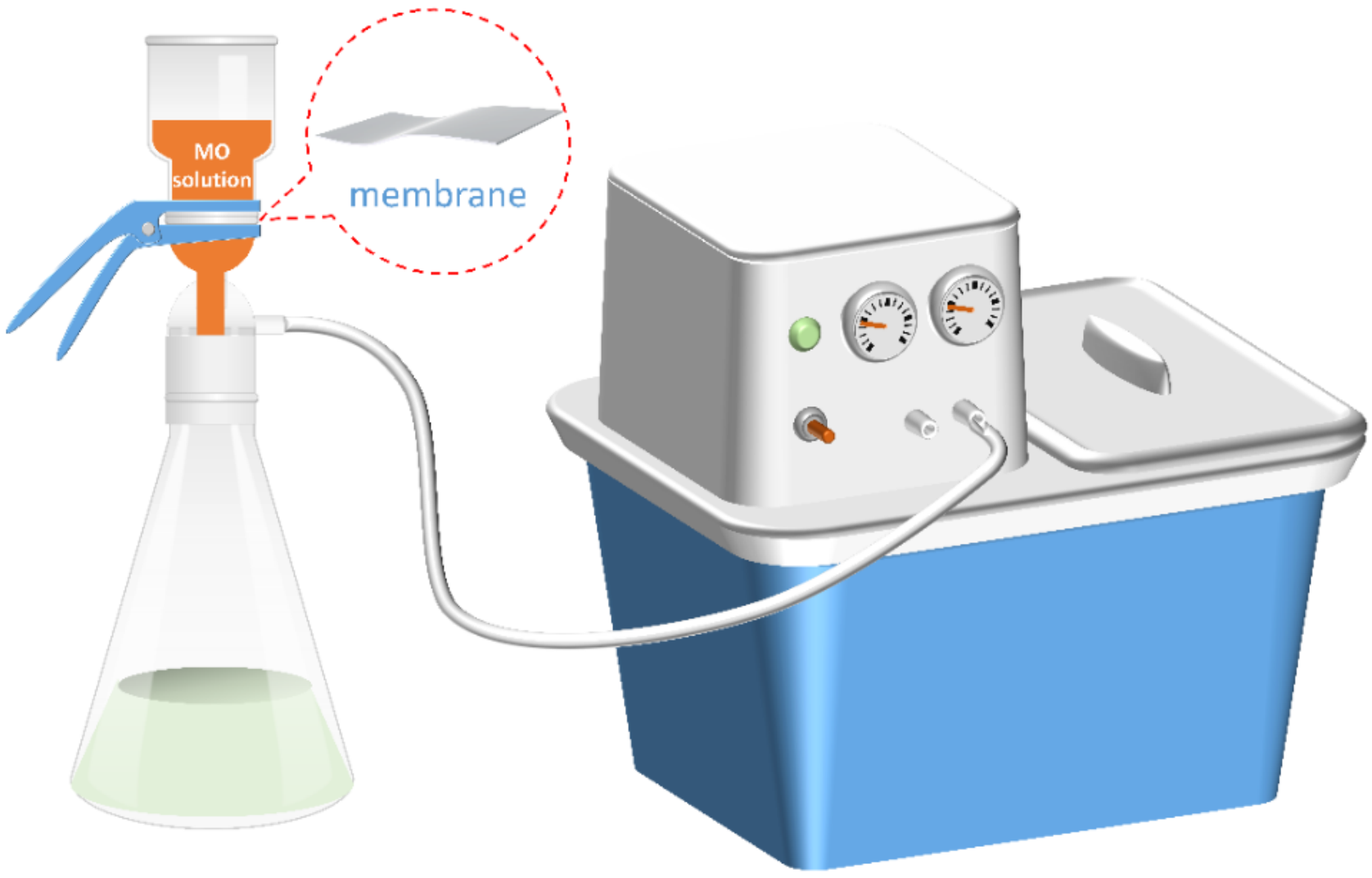

Fig. S18 Schematic illustration of the membrane filtration setup for MO removal.

13. Cycling test results of MOF@PES-2

Table S8 Cyclic adsorption–desorption performance of the membrane at an initial MO volume of 10 mL.

Cycle	Replicate 1 (%)	Replicate 2 (%)	Replicate 3 (%)	Mean (%)	SD
1st	98.85	97.50	95.90	97.42	1.48
2nd	95.46	88.54	93.92	92.64	3.63
3rd	87.58	89.81	80.00	85.80	5.15

Table S9 Cyclic adsorption–desorption performance of the membrane at an initial MO volume of 25 mL.

Cycle	Replicate 1 (%)	Replicate 2 (%)	Replicate 3 (%)	Mean (%)	SD
1st	98.91	96.52	93.98	96.47	2.46
2nd	96.44	78.77	90.84	88.68	9.03
3rd	75.46	22.13	14.84	37.48	33.10

14. Characterization of MOF@PES-2 after cyclic experiments

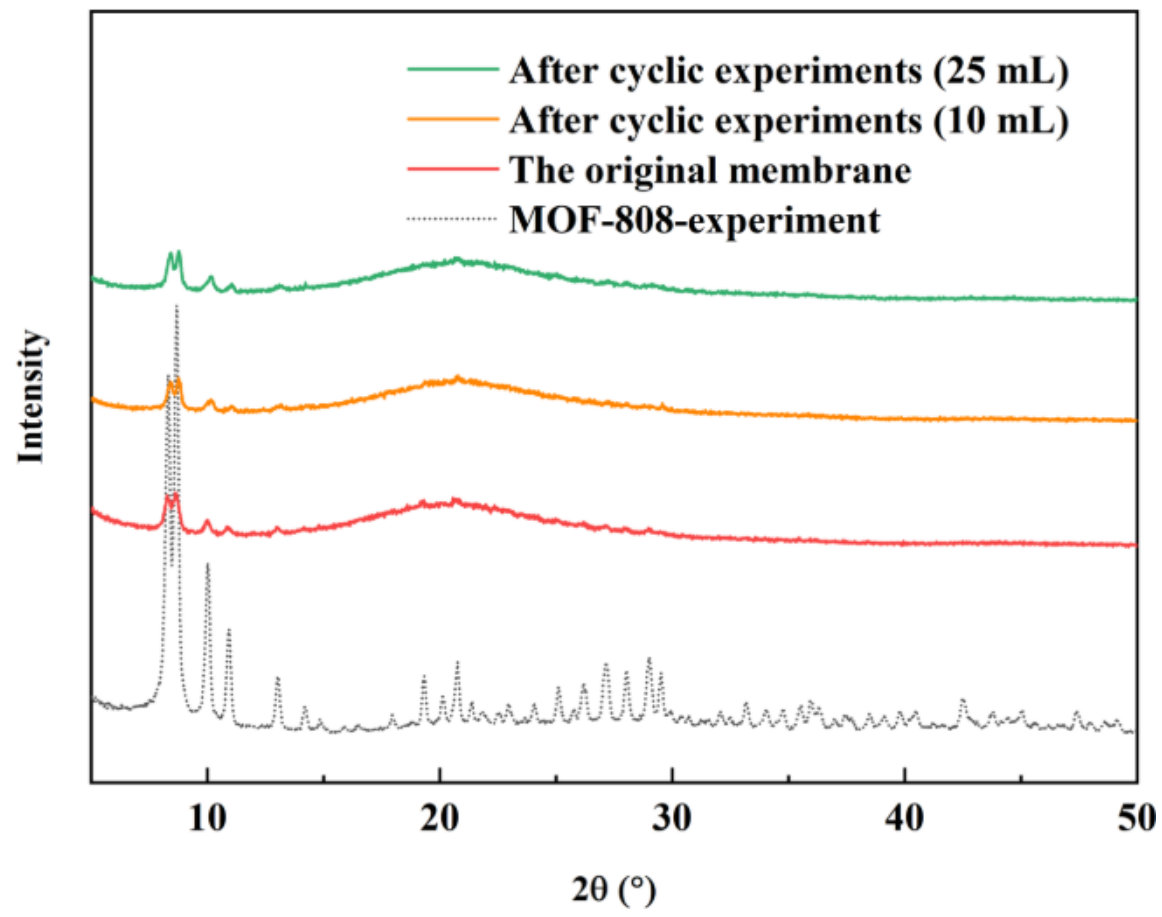

Fig. S19 PXRD patterns of MOF@PES-2 after cyclic experiments.

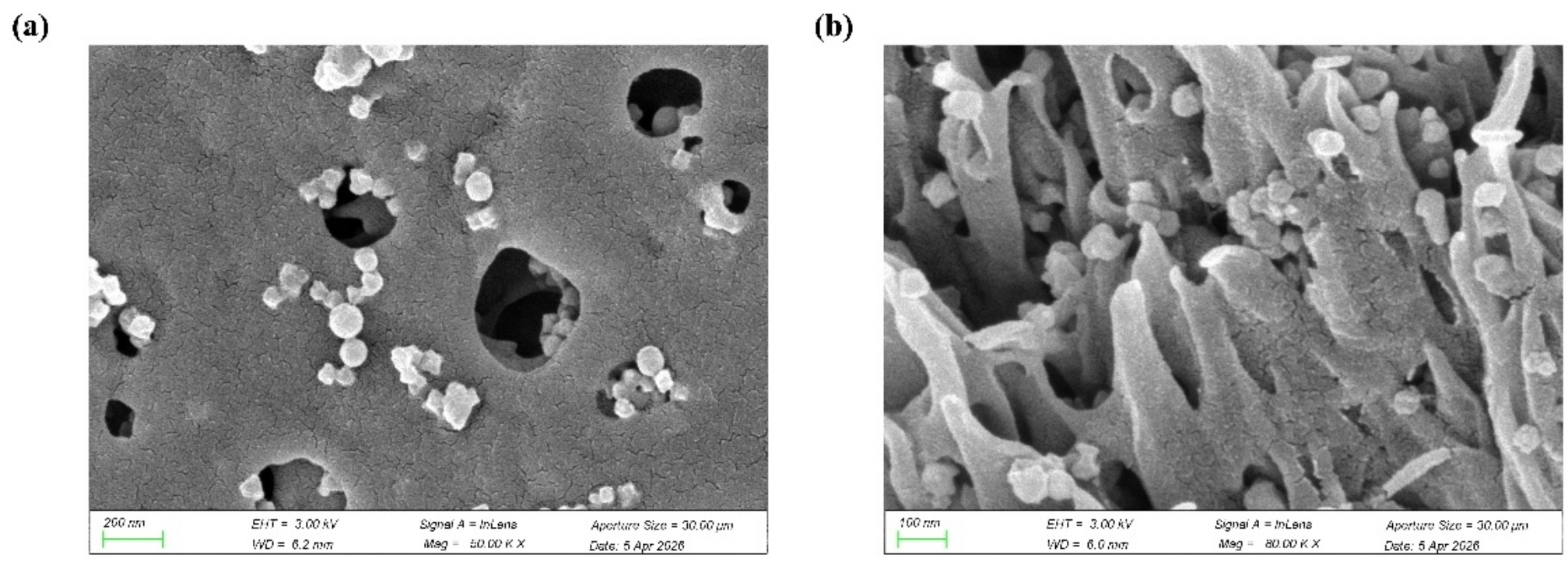

Fig. S20 Surface (a) and cross-sectional (b) SEM images and of MOF@PES-2 after MO filtration (C₀ = 30 mg L⁻^{1}, V = 10 mL).

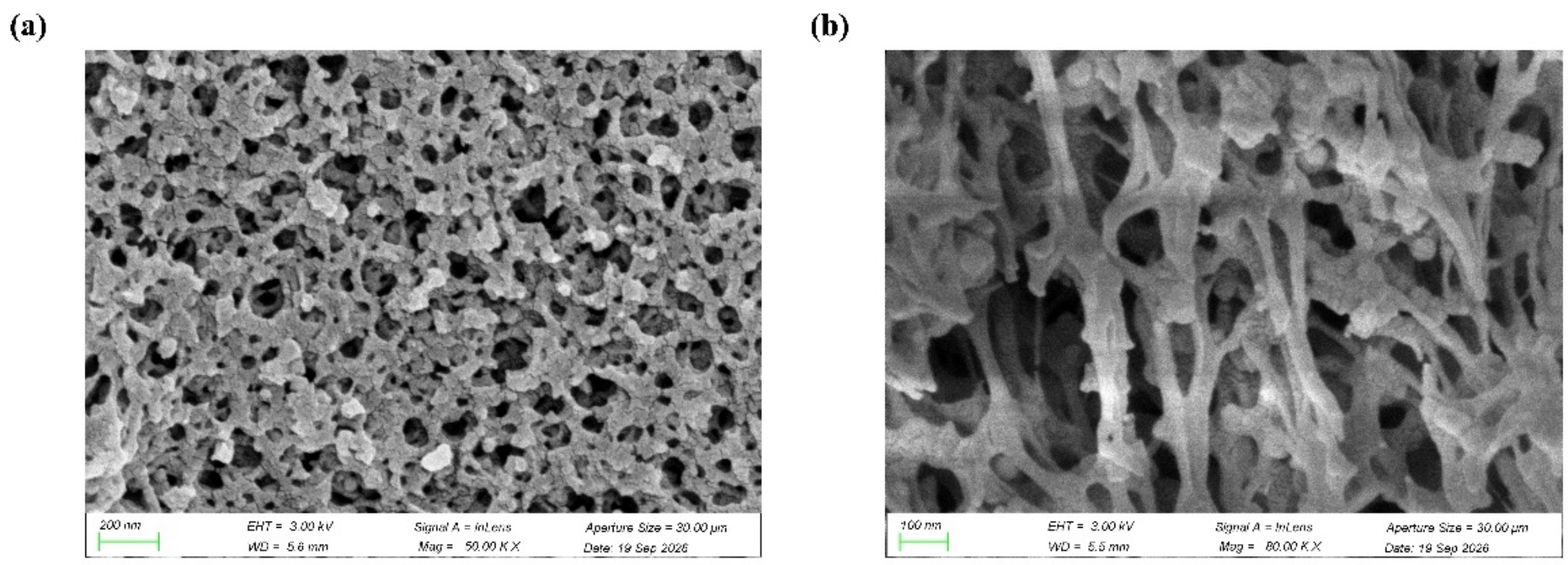

Fig. S21 Surface (a) and cross-sectional (b) SEM images of MOF@PES-2 after MO filtration (C₀ = 30 mg L⁻^{1}, V = 25 mL).

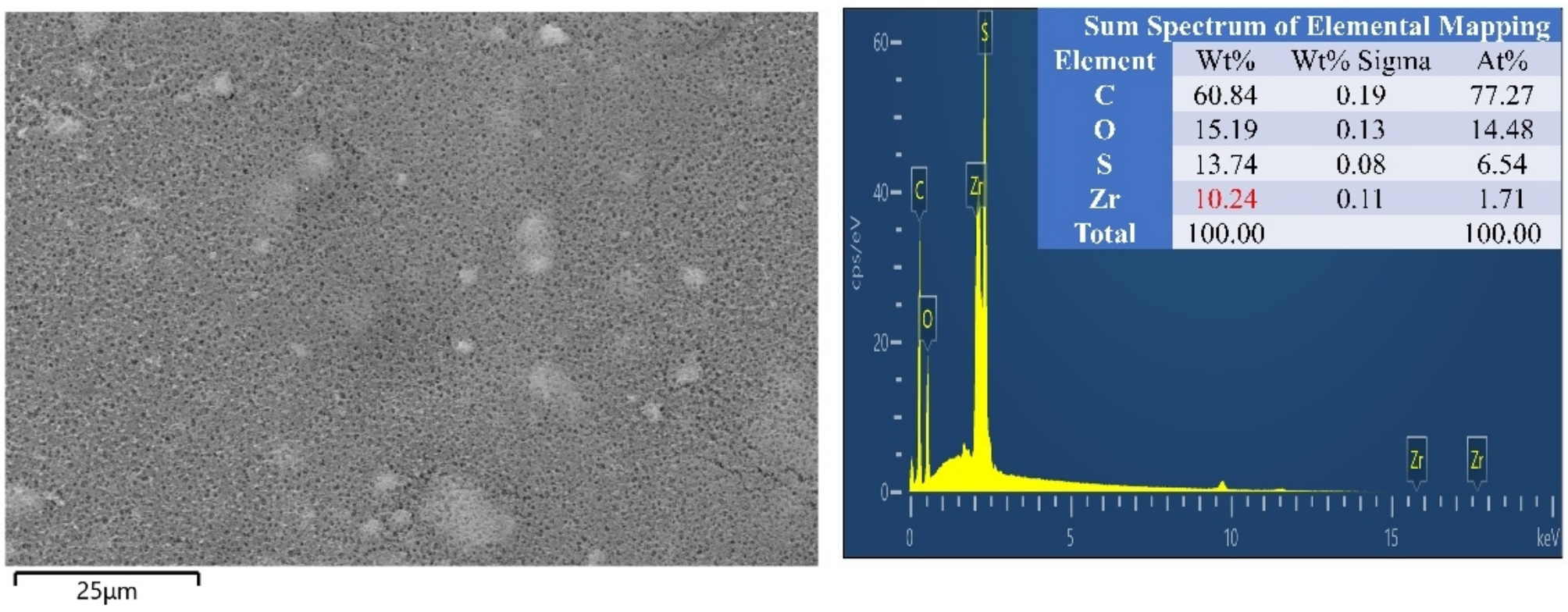

Fig. S22 EDS analysis of MOF@PES-2 before cyclic experiments.

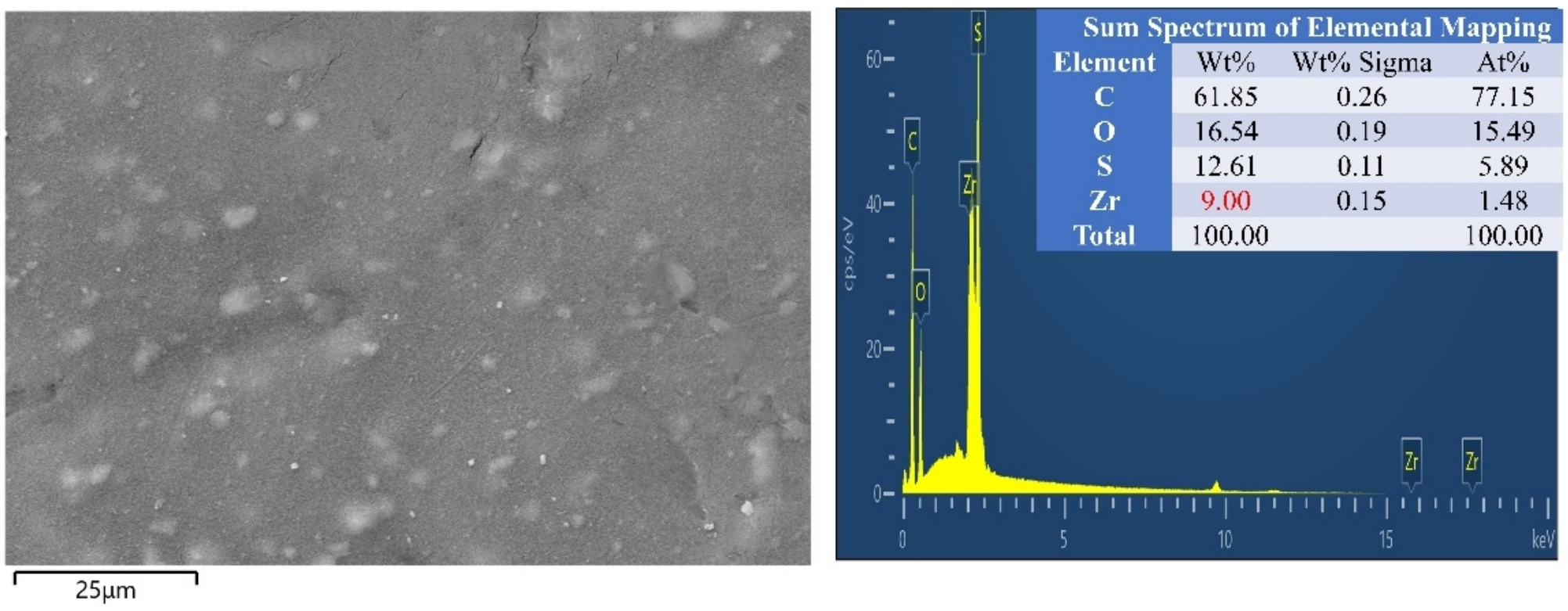

Fig. S23 EDS analysis of MOF@PES-2 after cyclic experiments (C_0 = 30 mg L^{-1}, V = 10 mL).

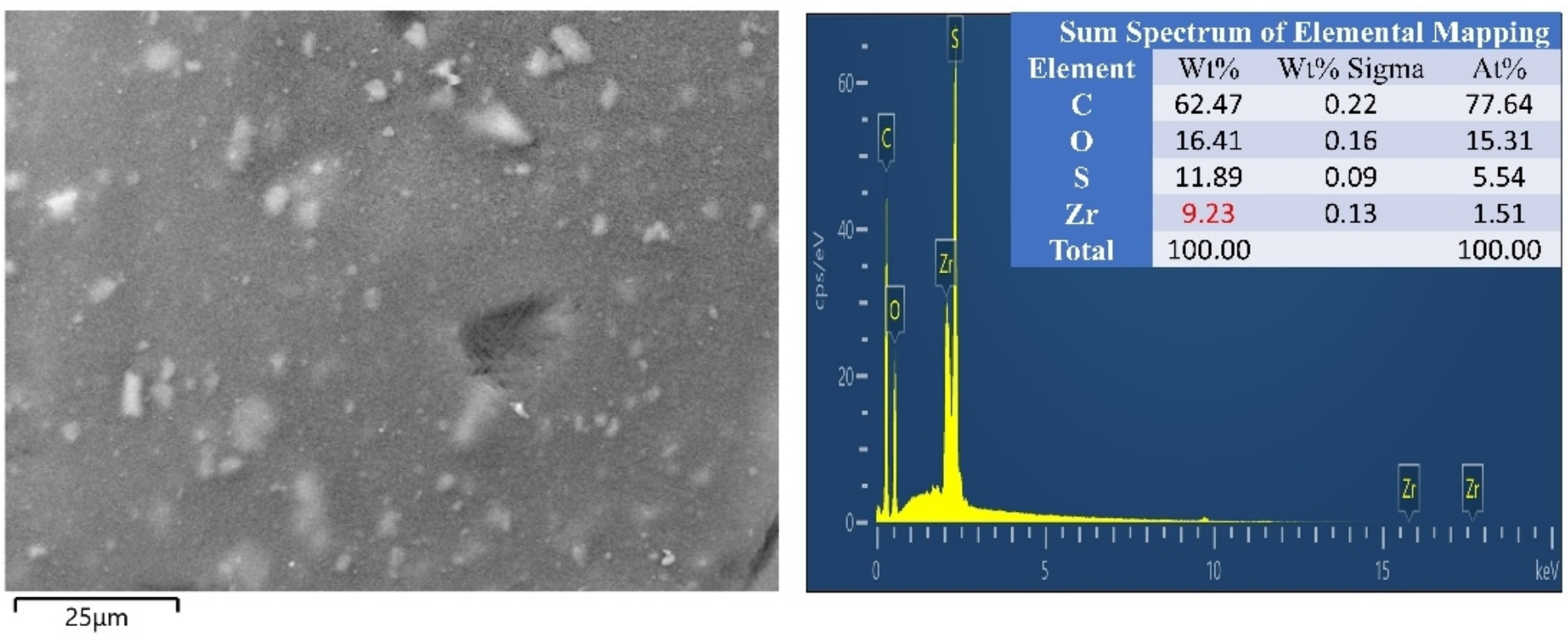

Fig. S24 EDS analysis of MOF@PES-2 after cyclic experiments (C_0 = 30 mg L^{-1}, V = 25 mL).

Table S10 Zr concentration in the filtrate after cycling tests (C_0 = 30 mg L^{-1}, V = 10 mL). determined by ICP-OES. *

Cycle	Zr concentration (mg/L)	Result	n
1st	n.d.	n.d.	3
2nd	n.d.	n.d.	3
3rd	n.d.	n.d.	3

*Each sample was measured in triplicate (n = 3). n.d. = not detected;

Table S11 Mechanical performance data for membranes after cycling tests (C_0 = 30 mg L^{-1}, V = 10 mL).

Samples number	Tensile strength/MPa	Elastic modulus/MPa	Elongation at break (%)
1	2.72	102.38	3.47
2	3.2	63.74	6.42
3	3.36	92.23	7.39
Mean	3.09	86.12	5.76
SD	0.33	20.03	2.04

Table S12 Mechanical performance data for membranes after cycling tests (C_0 = 30 mg L^{-1}, V = 25 mL).

Samples number	Tensile strength/MPa	Elastic modulus/MPa	Elongation at break (%)
1	4.97	150.43	5.5
2	4.19	118.04	5.03
3	4.33	145.8	5.36
Mean	4.50	138.09	5.30
SD	0.42	17.52	0.24

15. Characterization of MOF@PES-2 after filtration of NaCl-containing MO solutions

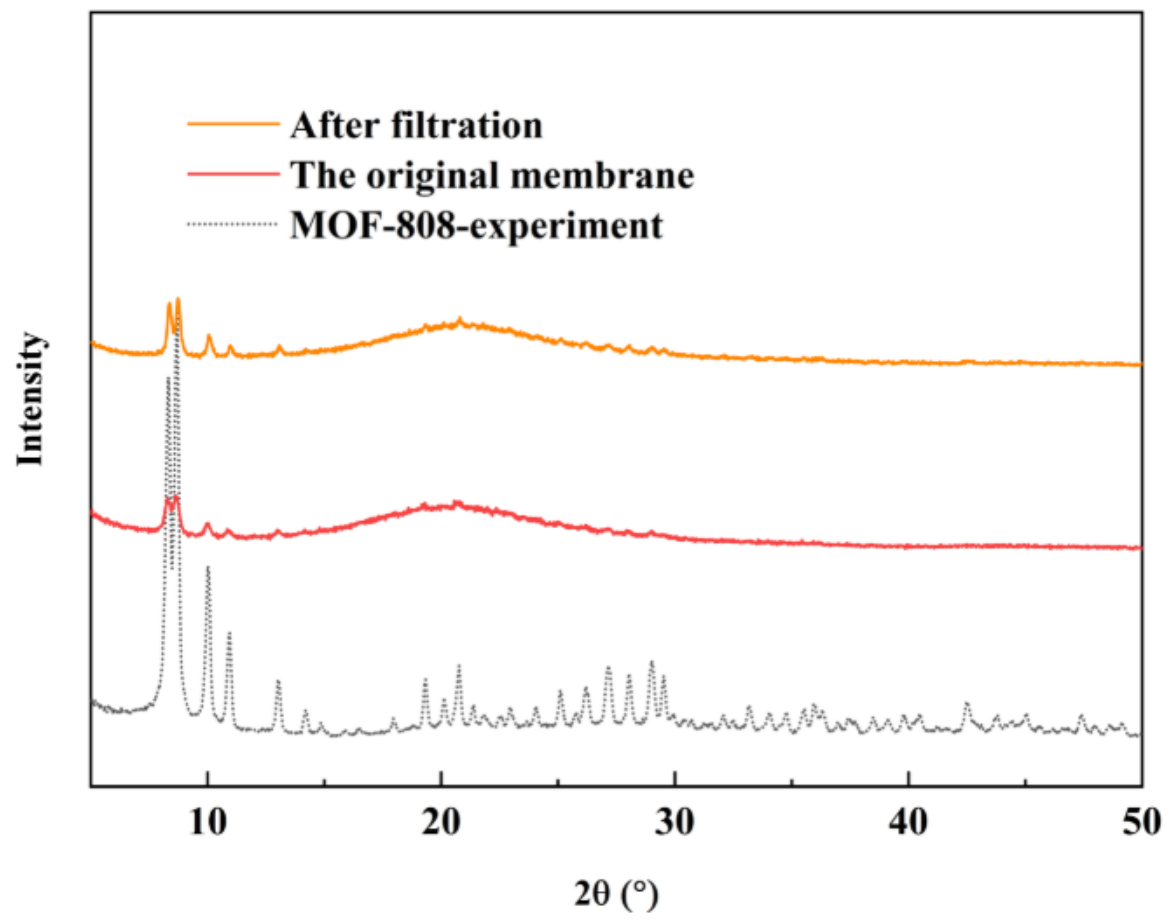

Fig. S25 PXRD patterns of MOF@PES-2 after filtration of a 300 mg L^{-1} NaCl-containing MO solution (MO: 30 mg L^{-1}, 10 mL).

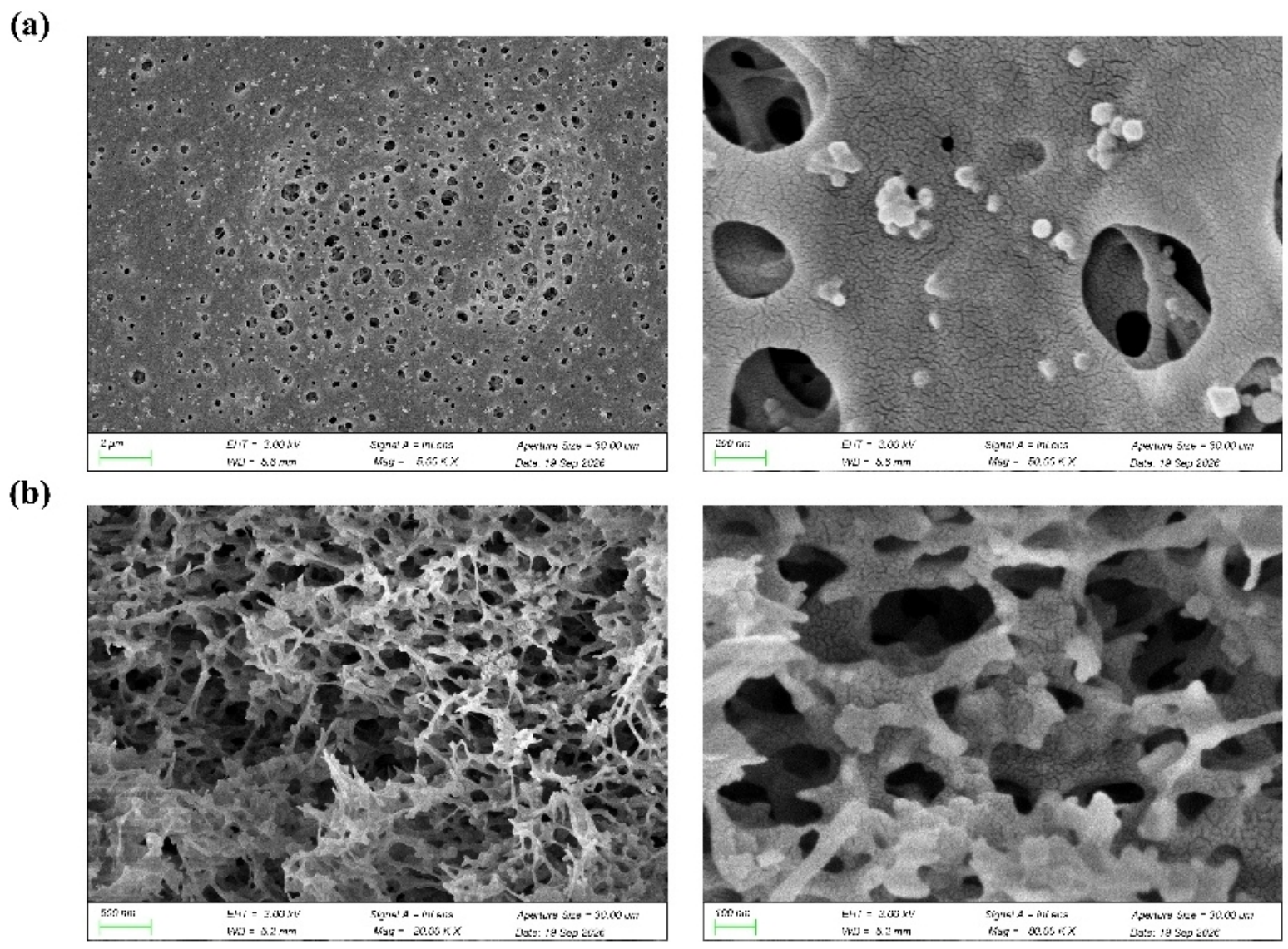

Fig. S26 Surface (a) and cross-sectional (b) SEM images and of MOF@PES-2 after filtration of a 300 mg L^{-1} NaCl-containing MO solution (MO: 30 mg L^{-1}, 10 mL).

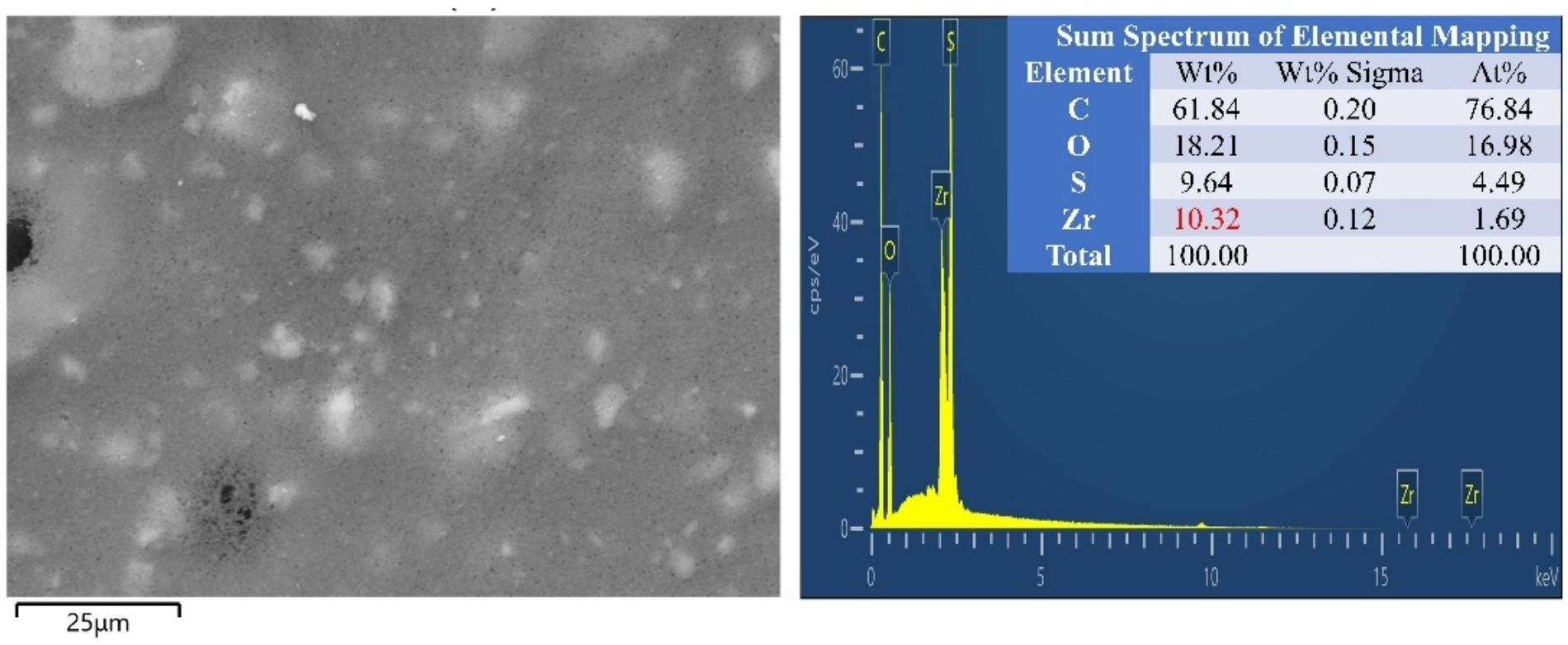

Fig. S27 EDS analysis of MOF@PES-2 after filtration of a 300 mg L^{-1} NaCl-containing MO solution (MO: 30 mg L^{-1}, 10 mL).

Table S13 Zeta potentials of MOF-808 in NaCl solutions with different concentrations.

NaCl solutions (mg L^{-1})	Mean (mV)	SD	n
0	32.0	1.13	3
30	30.03	1.44	3
300	26.77	0.57	3
3000	14.77	0.67	3

Table S14 Mechanical performance data for MOF@PES-2 after filtration of a 300 mg L^{-1} NaCl-containing MO solution.

Samples number	Tensile strength/MPa	Elastic modulus/MPa	Elongation at break (%)
1	4.36	243.99	3.05
2	3.6	178.37	2.78
3	4.65	230.96	3.45
Mean	4.20	217.77	3.09
SD	0.54	34.74	0.34

16. Comparison with recent literature for methyl orange removal

Table S15 Comparison of operational stability and reusability of MOF-based membranes for MO removal.

Membrane material system	MO removal rate/capacity	Number of cycles	Performance retention	Testing conditions	Reference
Al-UiO-66/PVDF MMM	>99%	10	>90%	Cyclic adsorption–desorption	11
Zwitterionic ZIF-8/PAN-PVP	98.9%	10	~93%	Continuous cycles, varying pH	12
PVC/MIL-101-NH₂	~29%	Not reported	/	Cross-flow filtration	13
MIL-101(Fe)/NiFe-LDH	91%	5	>80%	Cyclic filtration, FRR > 90%	14
MOF-808@PES-2	>97% (10 mL, 3 cycles); >96% (25 mL, 2 cycles)	2~3	85.80% (10 mL); 88.68% (25 mL)	Cyclic adsorption–desorption; 10–25 mL MO (30 mg/L)	This work
MOF-808@PES-2	>95% (equal-mass NaCl, 1 cycle); >89% (10× mass NaCl, 1 cycle)	1	/	Cyclic adsorption–desorption; 10 mL MO (30 mg/L); NaCl added	This work

17. References

1. H. Furukawa, F. Gándara, Y.-B. Zhang, J. Jiang, W. L. Queen, M. R. Hudson and O. M. Yaghi, J. Am. Chem. Soc., 2014, 136, 4369-4381.
2. X. J. Hu, Y. L. Li, H. X. Liu, S. M. Ying, Q. Yin and T. F. Liu, RSC Adv, 2024, 14, 12142-12146.
3. Z. Hasan, N. A. Khan and S. H. Jhung, Chem. Eng. J., 2016, 284, 1406-1413.
4. S. Zhuang, R. Cheng and J. Wang, Chem. Eng. J., 2019, 359, 354-362.
5. A. Karami, R. Sabouni and M. Ghommem, J. Mol. Liq., 2020, 305, 112808.
6. W. Hu, Q. Yang, R. Wang, Q. Chen and Y. Kong, 2025, 44, e14543.
7. L. Shen, F. Guo, Y. Hang, J. Yang, Z. Guo, W. Liang, P. Du and W. Jiao, J. Phys. Chem. Solids, 2025, 198, 112452.
8. M. Jannah, A. R. Putra Hidayat, F. Martak and R. Ediati, IOP Conf. Ser.: Earth Environ. Sci., 2024,

1388.
9. A. R. P. Hidayat, L. L. Zulfa, N. Faaizatunnisa, D. Prasetyoko, D. Hartanto, N. Widiastuti, A. S. Purnomo, M. Jannah, E. N. Kusumawati, H. Bahruji and R. Ediati, Nano-Struct. Nano-Objects, 2024, 37, 101077.
10. K. Wang, Y. Jiang and N. Yin, Process Saf Environ, 2026, 217, 109405.
11. P. Liu, J. Lyu and P. Bai, Sep. Purif. Technol., 2025, 354, 128672.
12. A. Zhengis, A. Ospanova, Y. Amrenova, P. Askar, A. Aldongarov, M. Karibayev, A. Aliyev, M. Abutalip and N. Nuraje, ACS Appl. Mater. Interfaces, 2025, 17, 65048-65062.
13. A. H. Hamzeh, S. M. Nezhad, E. Nazarzadeh Zare, J. Mahmoudi and H. Karimi-Maleh, J. Sci.:Adv. Mater. Devices, 2026, 11, 101102.
14. Shehriyar, A. Khurram, A. Jabbar, M. A. Khan, W. Liu, S. Mutahir, A. Munir and S. I. Al-Saeedi, Desalination, 2026, 617, 119438.
